# Exploiting light chains for the scalable generation and platform purification of native human bispecific IgG

**DOI:** 10.1038/ncomms7113

**Published:** 2015-02-12

**Authors:** Nicolas Fischer, Greg Elson, Giovanni Magistrelli, Elie Dheilly, Nicolas Fouque, Amélie Laurendon, Franck Gueneau, Ulla Ravn, Jean-François Depoisier, Valery Moine, Sylvain Raimondi, Pauline Malinge, Laura Di Grazia, François Rousseau, Yves Poitevin, Sébastien Calloud, Pierre-Alexis Cayatte, Mathias Alcoz, Guillemette Pontini, Séverine Fagète, Lucile Broyer, Marie Corbier, Delphine Schrag, Gérard Didelot, Nicolas Bosson, Nessie Costes, Laura Cons, Vanessa Buatois, Zoe Johnson, Walter Ferlin, Krzysztof Masternak, Marie Kosco-Vilbois

**Affiliations:** 1 Novimmune SA, 14 chemin des Aulx, CH-1228 Plan-les-Ouates, Switzerland

## Abstract

Bispecific antibodies enable unique therapeutic approaches but it remains a challenge to produce them at the industrial scale, and the modifications introduced to achieve bispecificity often have an impact on stability and risk of immunogenicity. Here we describe a fully human bispecific IgG devoid of any modification, which can be produced at the industrial scale, using a platform process. This format, referred to as a κλ-body, is assembled by co-expressing one heavy chain and two different light chains, one κ and one λ. Using ten different targets, we demonstrate that light chains can play a dominant role in mediating specificity and high affinity. The κλ-bodies support multiple modes of action, and their stability and pharmacokinetic properties are indistinguishable from therapeutic antibodies. Thus, the κλ-body represents a unique, fully human format that exploits light-chain variable domains for antigen binding and light-chain constant domains for robust downstream processing, to realize the potential of bispecific antibodies.

## Abstract (web summary)

Bispecific antibodies allow for novel therapeutic approaches but industrial-scale production and immunogenicity represent significant challenges. Here Fischer *et al.* describe a unique human bispecific antibody format that exploits differing light chains to overcome these obstacles.

## Graphical abstract

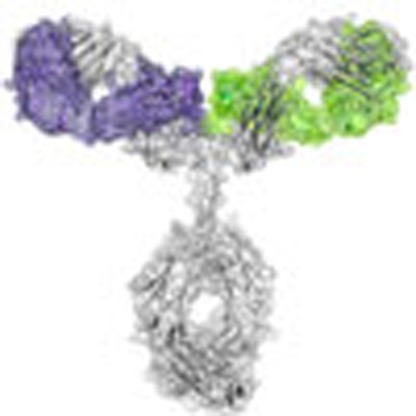

Antibodies are characterized by two functionally important regions, the Fab (two of which are present in a single antibody molecule) and the Fc, the former dictating target specificity and the latter influencing effector function as well as half-life *in vivo*. Over 30 monoclonal antibodies (mAbs) are marketed as medicines, providing physicians with powerful approaches to effectively treat a diverse range of medical conditions^1^. This success is explained in part by the exquisite specificity of mAbs, as the two Fab fragments typically bind only one target, thereby providing minimal off-target side effects, resulting in low intrinsic toxicity. A second fundamental factor is the existence of scalable manufacturing processes that can be applied in a globally generic manner to mAbs, thus substantially facilitating clinical and commercial development.

Bispecific antibodies (BiAbs) represent a new dimension in terms of therapeutic potential, while exploiting the beneficial structural characteristics of mAbs. BiAbs generally build on the feature of having two antigen-binding sites (Fab or single-chain Fv (scFv)), each one used to provide a different target-binding specificity, albeit within the same molecule. This configuration enables a multitude of novel mechanisms of action that cannot be mediated by mAbs, such as targeting of immune cells to tumours, co-engagement of receptors, increased transport across the blood–brain barrier and replacement of missing coagulation factors^2^,^3^,^4^,^5^. The clinical efficacy and market approval of Blinatumomab^6^,^7^ and Catumaxomab^8^ provide therapeutic validation of BiAbs, thus intensifying efforts to develop optimal BiAb formats. Interestingly, many methods alter the native antibody sequence by adding foreign sequences (that is, integrating linkers to connect antibody fragments), surface remodelling (that is, mutating the interfaces between Fc domains to promote molecular assembly) or engineering novel binding sites into Fc domains^9^,^10^,^11^,^12^,^13^. These alterations often have a significant negative impact on expression yield and product stability, as well as an increased potential for provoking anti-drug responses in patients. As a result, manufacturing at scales relevant for the clinic is often a major hurdle to the development of BiAbs^14^,^15^.

Given the level of accumulated experience with mAbs, an appealing bispecific format for therapeutic use would be an unmodified human IgG. This format would share stability, pharmacokinetic and other sought-after drug-like properties of therapeutic mAbs, while enabling novel modes of action. An approach previously tried was to co-express the heavy and light chains of two different antibodies in a single cell. However, the random assembly of the four chains resulted in a complex mixture of ten molecules, substantially challenging development from yield, cost and purity perspectives^9^. A more selective approach is to use antibodies that share a common chain such that concomitant expression of two heavy and a common light chain in the same cell results in a mixture containing only two mAbs and one BiAb. However, the downstream purification of the BiAb from this mixture is challenging and relies on differences between the physicochemical properties (for example, overall charge or hydrophobicity) of the BiAb and the two mAbs^16^.

Here we describe a novel technology platform to generate unmodified fully human BiAbs, exploiting a generic downstream purification process compatible with industrial-scale manufacturing. The strategy involves the creation of *in vitro* display libraries with common heavy chains that are used to select against two different antigens. This allows the isolation of candidates with different target specificities that share the same heavy chain but carry either κ or λ light chains. Three different chains (one heavy and two light) are then co-expressed in a single cell to generate a mixture containing two mAb species (one κ and one λ) and a BiAb containing a κ and λ light chain (Fig. 1). A BiAb assembled in this manner can then be efficiently purified from the mAb species and other contaminants using highly selective affinity resins binding to either human κ or λ constant domains. Based on its structure, this fully human BiAb format is referred to as a κλ-body.

## Results

### Generation of mAbs sharing a common heavy chain

**Figure 1:**
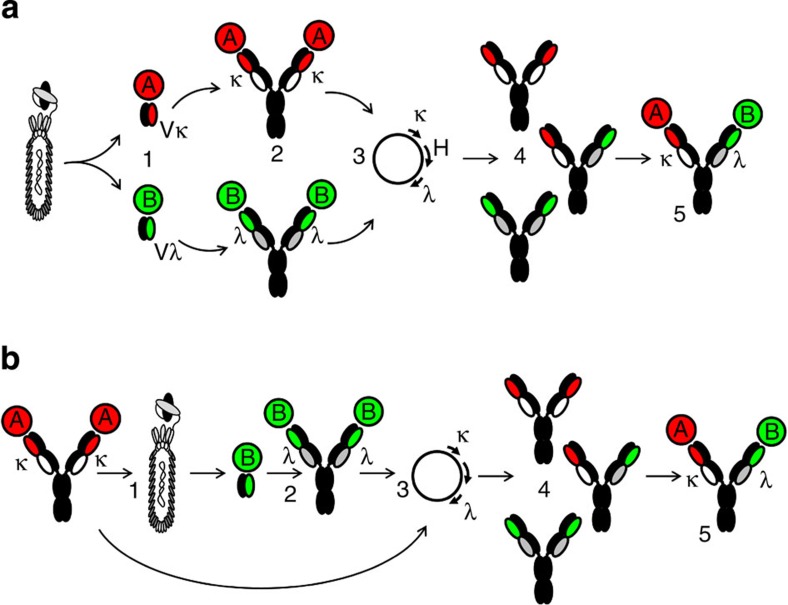
Approaches for the generation of bispecific IgG based on light-chain diversity. (**a**) Parallel discovery of two bispecific arms from a fixed VH library. (1) Phage-display scFv libraries containing a single VH and diversified VL are used for selection and screening of scFv specifically binding to two different proteins (A and B). The libraries containing κ and λ variable light-chain domains are kept separated. (2) scFv candidates are reformatted into IgG and characterized for binding and functional activity. (3) The common heavy chain and two light chains (one κ and one λ) are cloned into a single mammalian expression vector. (4) Co-expression of the three antibody chains leads to the expression and secretion of an antibody mixture with a theoretical distribution of 25% monospecific κ, 25% monospecific λ and 50% bispecific IgG with κ and λ light chains (κλ-body). (5) Bispecific κλ-bodies specific for target A and B are purified using affinity resins binding to constant regions of the heavy chains (either CH1 or Fc) and to the constant regions of the κ and λ chains. The affinity-purification process can be used for any arm combination (as described in Fig. 3). (**b**) Sequential discovery of a second arm compatible with an existing antibody. (1) The VH domain of an antibody directed against target A is combined with diversified variable light chains to build a scFv phage display library. If the first antibody contains a κ light chain, then diversified λ light chains are used to build the library, or vice versa. (2) The resulting library is used to identify scFv candidates against a second target, B, and are reformatted into IgG for characterization. Steps 3–5 are identical to those described for (**a**).

**Figure 2:**
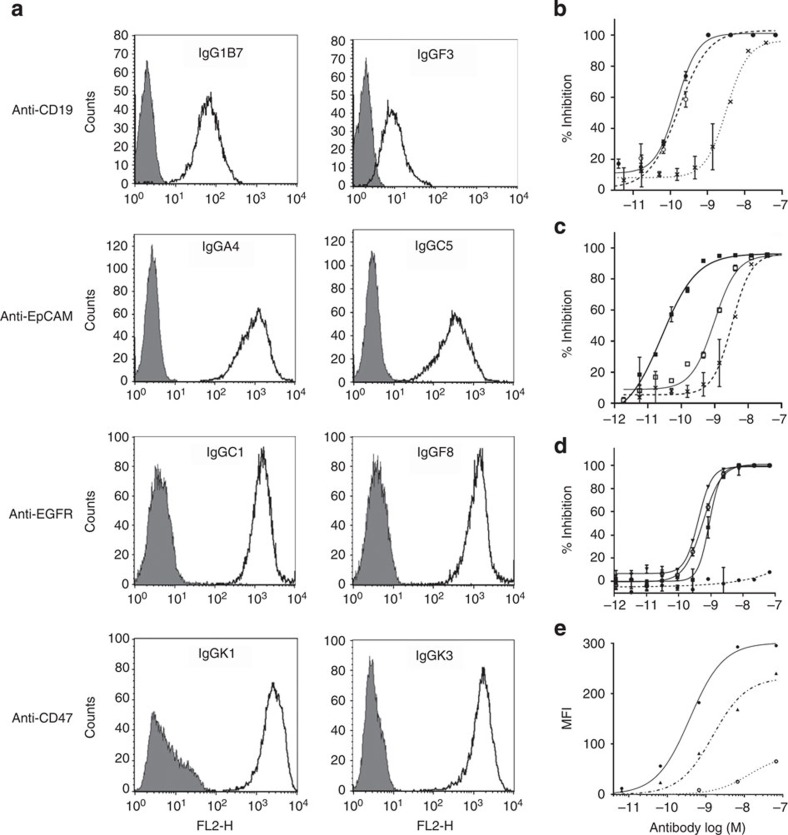
Common heavy-chain antibodies are specific and have neutralization potential. (**a**) Flow cytometry profiles obtained with different IgGs selected against CD19, EpCAM, EGFR and CD47. The profiles on antigen-negative versus -positive cell lines are shown shaded and open, respectively (for CD19: Jurkat/Raji; EpCAM: HEK-293/MCF-7; EGFR: MCF-7/A431; CD47: CHO/CHO-CD47). (**b**) Dose–response inhibition of SIRPα binding to CD47 expressed on cells for the positive control mAb B6H12 (open circles), IgGK1 (closed circles) and IgGK2 (crosses) that were directly isolated from the fixed heavy-chain libraries. (**c**) Neutralization potential in the SIRPα/CD47-binding assay for variants IgGK3 and IgGK4 (closed and open squares, respectively) obtained via optimization of IgGK2 (crosses). (**d**) Neutralization activity in the MSLN/MUC16 interaction assay for IgGO1 (open circles) and IgGO3 (closed triangles) in comparison with a negative control mAb (closed circles) and the therapeutic antibody Amatuximab (closed squares). (**e**) Dose–response binding profile measured by flow cytometry on CD19-positive Raji cells for IgG1B7 (open circles) and variants IgGL7-1 (triangles), and IgGL7-2 (closed circles) that were generated by affinity maturation. Error bars in **b**, **c** and **d** represent s.e.m. of two replicates. The data are representative of three independent experiments.

To enable the identification of mAbs binding with high specificity to two different antigens but having an identical heavy chain, we generated several human antibody variable gene repertoires combining a unique rearranged heavy chain variable gene (VH) and different repertoires of light chain variable genes (VL). We have used either generic fixed VH or the VH from an existing mAb. In the first case, the repertoire containing a generic VH can be used simultaneously for the *de novo* isolation of two antibodies sharing the same VH against two antigens (Fig. 1a). In the second approach, the VH of a first mAb is combined with a VL repertoire for the isolation of a second mAb with specificity for a second target (Fig. 1b). The VL sequences were either isolated from circulating B cells from healthy individuals or generated *in vitro* using different diversification strategies. A total of 15 scFv phage display libraries were built, containing either diversified λ or κ VL genes (Supplementary Table 1). In each library, the VL repertoire was combined with a unique rearranged VH based on the *IGHV3-23*, *IGHV3-30* or *IGHV3-48* germline genes^17^. These VH genes were chosen for their frequent occurrence in natural human antibody repertoires, intrinsic stability and suitability for *in vitro* display technologies^18^,^19^. The total diversities of the κ and λ libraries were 5.6 × 10^10^ and 1.6 × 10^10^, respectively. These fixed heavy chain libraries were used separately for phage display selection and screening campaigns against several soluble and cell surface human antigens, including CD47, CD19, EpCAM, EGFR, HER2, IFNγ, IL6R, MSLN, GPC3 and FOLR1. More than 680 individual sequences encoding scFvs specifically binding to one of these antigens were selected and confirmed to contain different κ and λ light chains. Out of the 173 unique sequences that were reformatted and expressed as hIgG1, 103 were confirmed for specific binding against their respective target antigen by fluorescence-activated cell sorting and enzyme-linked immunosorbent assay (ELISA; Table 1 and Fig. 2a). The binding affinities for several IgGs were determined by biolayer interferometry or surface plasmon resonance to be in the subnanomolar to the micromolar range (Supplementary Table 2). More than half of the candidates isolated directly out of the fixed VH libraries had affinities in the low nanomolar range, which is comparable to candidates isolated from standard scFv libraries bearing diversity in both heavy and light chains, thus indicating that light chains can efficiently drive high-affinity binding^20^,^21^.

The interaction between CD47 and its ligand, SIRPα (expressed on myeloid cells), conveys a negative signal that inhibits phagocytosis and plays an important role in the recognition of self by the immune system as well as other biological processes^22^,^23^. We therefore evaluated whether the anti-CD47 IgGs generated by this approach could block the interaction between these two proteins in an assay monitoring the binding of SIRPα on cells expressing CD47. Both IgGK1 and IgGK2 were able to neutralize this interaction in a dose-dependent manner. The subnanomolar IC_50_ observed with IgGK1 was similar to that of the neutralizing anti-hCD47 murine mAb, B6H12 (ref. 24; Fig. 2b). The interaction between MSLN and CA125/MUC16 promotes cancer cell dissemination and correlates with poor prognosis in patients^25^. We tested the neutralization activity of some of the anti-MSLN candidates and showed that the IgGO1 and IgGO3 could block the interaction between MSLN and CA125/MUC16 *in vitro*, with an IC_50_ similar to the therapeutic anti-MSLN mAb Amatuximab (Fig. 2d). Taken together, these results demonstrate that antibodies can be isolated directly from fixed heavy chain libraries with target–ligand neutralization capacity.

Eight candidates directed against either hCD19, hCD47 or hEpCAM were selected for affinity maturation by further diversification of their VL sequences. Different randomization strategies targeting the complementary determining regions (CDR) were applied and the resulting libraries were used for selections under increasingly stringent conditions. In all cases, scFvs with a higher affinity could be isolated and confirmed in an IgG format. For instance, variants of the anti-CD47 IgGK2 with up to a >100-fold increased neutralization potential in the CD47-SIRPα assay were generated (Fig. 2c). Another example is the low-affinity anti-CD19 IgG1B7 from which the improved variant IgGL7-1 was derived. The latter was then further optimized in a second affinity maturation round to obtain IgGL7-2, which had a 50-fold increase in affinity over the parental antibody and, subsequently, a significantly improved binding on CD19^+^ Raji cells (Fig. 2e and Supplementary Table 2). This data demonstrates that the affinity of antibodies initially isolated from fixed heavy chain libraries can be increased by optimization of the VL, while leaving the VH unmodified throughout the selection process. Taken together, the generation of a panel of over 100 human IgGs sharing a common VH and directed against 10 different antigens confirms that the light chain is sufficient to drive antibody specificity and enables the isolation of high-affinity antibodies with neutralizing activity.

### Assembly and purification of κλ-bodies

**Figure 3:**
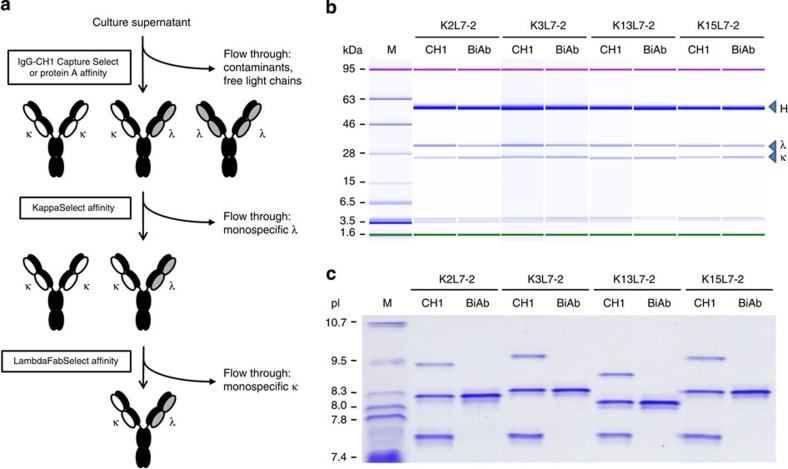
Purification and characterization of κλ-bodies. (**a**) Purification workflow. In the first step, total IgGs are recovered from the culture supernatant either using protein A or IgG-CH1 Capture Select affinity chromatography, resulting in the elimination of free light chains and other contaminants. In the second step, IgG containing a κ light chain are captured using KappaSelect affinity resin and the monospecific IgGλ are eliminated in the column flow through. In the final step, pure bispecific κλ-bodies are recovered using LambdaFabSelect affinity resin and separated from the monospecific IgGκ that do not bind to the resin. (**b**) Analysis on an Agilent Bioanalyzer 2100 of total IgG obtained after the first IgG-CH1 Capture Select affinity chromatography step (CH1) and after the third affinity chromatography steps (BiAb) for several κλ-bodies. The bands corresponding to the common heavy chain (H) and the κ and λ light chains are indicated. (**c**) Analysis of the same fractions by isoelectric focusing. The top and bottom bands observed after IgG-CH1 Capture Select affinity chromatography step correspond to the two mAbs, whereas the central bands (that is, with an intermediate isoelectric focusing point) correspond to the κλ-body.

The Vκ, Vλ and common VH sequences of two mAbs to be combined into a κλ-body were cloned into a single expression vector containing three expression cassettes, one for each of the κ and λ light chains (the latter containing the IGLC2λ constant region) and one for the common IgG1 heavy chain. Each cassette was under the transcriptional control of the human cytomegalovirus promoter. Overall, the method was tested with the VH and VL of 19 IgGκ and 10 IgGλ for the expression of 51 κλ-bodies (Supplementary Table 3). Small-scale transient transfections were performed in PEAK cells, leading to the production of three species: an IgGκ, an IgGλ and the κλ-body, with a theoretical distribution of 25%, 25% and 50%, respectively, provided the expression and assembly of the three chains is equivalent. κλ-bodies were then purified from the supernatant by a three-step affinity process (Fig. 3a). First, a non-distinguishing affinity-capture chromatography step was performed using either a protein A or Capture Select CH1 resin that binds to the Fc or CH1 domain of human IgG, respectively. The common heavy chain and the two light chains differ in molecular weight and therefore could be observed by gel electrophoresis (Fig. 3b and Supplementary Fig. 1a). Furthermore, the actual distribution of three IgG forms in the supernatant was evaluated after this first purification step by separation on an isoelectric focusing gel (Fig. 3c and Supplementary Fig. 1b). The total IgG yield (8–52 μg ml^−1^) was in a range similar to those obtained for standard mAbs using this transient expression system (Supplementary Table 3), indicating that the expression of three chains in this manner did not have a negative impact on overall IgG expression. The second affinity-capture purification step, using the KappaSelect affinity resin, which binds to the human κ constant region, allows for the recovery of the IgGκ and the κλ-body, while the IgGλ is eliminated in the column flow through. Finally, the κλ-body is purified to homogeneity by a third affinity-capture step, using the LambdaFabSelect affinity resin that binds selectively to human λ constant region, thus eliminating the IgGκ species (Fig. 3c). The purified κλ-bodies were generally low in aggregate content (average of 2.8%) and in a range similar to that found for human mAbs expressed using this expression system (Supplementary Table 3). Thus, these three affinity steps enable a fast, robust and generic purification process that was successfully applied to the 51 κλ-bodies characterized in this study.

### Receptor co-engagement and T-cell retargeting

**Figure 4:**
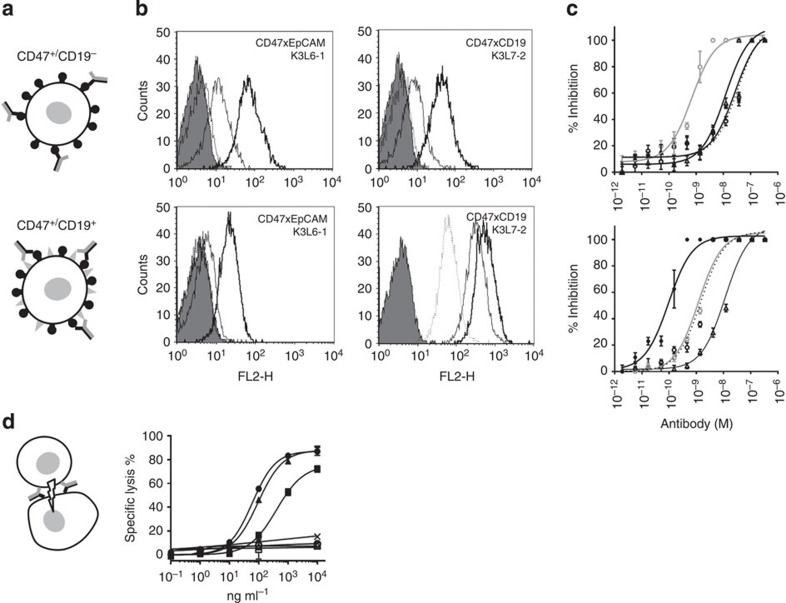
Biological activity of bispecific κλ-bodies. (**a**) Schematic representation of monovalent and bivalent binding of CD19xCD47 κλ-bodies on the surface of CD47^+^/CD19^−^DS-1 cells (top) and CD47^+^/CD19^+^Raji cells (bottom). (**b**) Binding of CD47 × EpCAM (left panels) and CD47 × CD19 κλ-bodies (right panels) to DS-1 (top panels) and Raji cells (bottom panels) monitored by FACS. κλ bodies were used at 0.01, 0.1 and 1 mg ml^−1^, control staining with fluorescently labelled secondary antibody is indicated by the shaded area. (**c**) Inhibition of SIRPα binding to the CD47 on DS-1 (top) and Raji cells (bottom) by increasing concentrations of different antibodies: anti-CD47-positive control mAb B6H12 (grey open circles); CD47 × CD19 K3L7-1 (black open circles, dotted line); CD47 × CD19 K3L7-2 (closed circles); CD47 × EpCAM K3L6-1 (open triangles). Error bars represent s.e.m. of four replicates and the data are representative of three independent experiments. (**d**) T-cell-mediated killing of EpCAM-positive cells by CD3 × EpCAM κλ-bodies. MCF-7 cells were incubated with purified human T cells in the presence of increasing concentrations of different antibodies. CD3 × EpCAM κλ-bodies: L6-3L3-1 (closed circles), L6-4L3-1 (closed squares), L6-5L3-1 (closed triangles); anti-EpCAM mAbs: L6-3 (open circles), L6-4 (open squares), L6-5 (open triangles); anti-CD3 mAb L3-1 (crosses). Error bars represent s.d. of three replicates and the experiment was repeated with the blood of two independent donors. The isoelectric focusing (IEF) gels and electrophoresis profiles corresponding to the different κλ-bodies and mAbs are shown in Fig. 3 and Supplementary Fig. 1.

Inhibition of the CD47-SIPRα interaction has been demonstrated to be an attractive strategy to increase the phagocytic activity of an anti-CD20 mAb, whereby phagocytosis was significantly increased by the co-injection of an anti-CD47 mAb, leading to complete tumour eradication^24^. Although appealing, targeting CD47 with a mAb is challenging due to the ubiquitous expression of this receptor. Selective inhibition of the CD47-SIRPα interaction on tumour cells is potentially achievable using a BiAb having one arm targeting a tumour-associated antigen and the other arm targeting CD47. The co-engagement of the two receptors should subsequently lead to increased binding on cells expressing the tumour-associated antigen via an avidity effect. We evaluated this possibility by testing the capacity of two CD47 × CD19 κλ-bodies to bind to the surface of CD19^+^ B cells (Raji) versus CD19^−^ B cells (DS-1; Fig. 4a). Raji cells express similar levels of CD47 and CD19 (65,000 and 72,000, respectively) and 136,000 CD47 molecules are found at the surface of DS-1 cells, while neither Raji nor DS-1 cells express EpCAM (Supplementary Table 4). Binding of K3L7-2 to the surface of Raji cells was increased compared to DS-1 cells, indicating that CD19 and CD47 are co-engaged by K3L7-2 on Raji cells. In contrast, the CD47 × EpCAM κλ-body, K3L6-1, can only bind via the anti-CD47 K3 arm on either cell type, as neither express EpCAM. The monovalent engagement of K3L6-1 leads to weaker binding to both cells types (Fig. 4b). We then compared the potency of the anti-CD47 mAb, B6H12, with K3L7-1 and K3L7-2, as well as the K3L6-1 (that is, the monovalent control), to inhibit the binding of SIRPα to CD47 on the surface of Raji and DS-1 cells. As expected, the IC_50_ observed with the anti-CD47 mAb is equivalent (1.2 × 10^−9^ and 0.7 × 10^−9^ on Raji and DS-1 cells, respectively; Fig. 4c). The potency of K3L6-1 was similar on both cell types, but lower compared with the CD47 mAb due to a loss of avidity (IC_50_ of 1.1 × 10^−8^ and 1.2 × 10^−8^ on Raji and DS-1 cells, respectively). In contrast, the potency of the CD19 × CD47 κλ body K3L7-1 was more than tenfold higher on Raji cells than on DS-1 cells (IC_50_ of 1.4 × 10^−9^ and 2.6 × 10^−8^, respectively). Moreover, the CD19 × CD47 κλ body K3L7-2 that incorporates the higher-affinity CD19 arm L7-2 was 300-fold more potent at inhibiting SIRPα binding on Raji than on DS-1 cells (IC_50_ of 10^−10^ and 3.3 × 10^−8^, respectively; Fig. 4c). These experiments demonstrate that CD19 × CD47 κλ-bodies are able to co-engage CD47 and CD19 on the surface of Raji cells, and that the selectivity for CD19^+^ cells can be fine-tuned by modifying the affinity of the κλ-body arms.

Tumour cell killing via retargeting of T cells with an anti-CD3 containing BiAb is an extensively studied and clinically validated mechanism of action^6^,^8^,^26^,^27^. To exemplify the utility of the κλ-body in additional modes of action, we generated CD3 × EpCAM candidates. In this case, we used the sequential discovery approach (Fig. 1b) and used an existing anti-CD3 mAb as a starting point. We combined its VH (germline *IGHV3-48*) with repertoires of diversified light chains, to generate a library of 9.5 × 10^9^ transformants (Library 48K, Supplementary Table I). This library was then used for phage display selections and lead to the isolation of candidates specific for EpCAM (Table I). As the resulting IgGs share the same VH as the starting anti-CD3 mAb, they could directly be used to generate CD3 × EpCAM κλ-bodies by co-expression of the two light chains and the common heavy chain as described above. Three of these κλ-bodies, that is, L6-3L3-1, L6-4L3-1 and L6-5L3-1, were tested in a T-cell retargeting assay, using T cells purified from human blood and MCF-7 cells expressing EpCAM as target cells. Only the CD3 × EpCAM κλ-bodies were able to mediate MCF-7 killing in a dose-dependent manner, while the anti-CD3 and anti-EpCAM mAbs were inactive (Fig. 4d). Thus, via monovalent engagement of two different antigens either in *cis* at the surface of the same cell or in *trans* on two different cells, κλ-bodies exhibit mechanisms of action that are unique to BiAbs.

### Manufacturability of κλ-bodies

We next investigated the industrial applicability of our platform manufacturing process design using two model κλ-body candidates, H2-1H1-1 and H2-1H2-2. To this end, we generated κλ-body-expressing recombinant GS-CHO cell lines for each product using the same ‘triple-gene’ expression vectors used for the transient transfections described above. Each cassette was under the transcriptional control of the cytomegalovirus promoter. In parallel, for κλ-body, H2-1H1-1, cell lines expressing monospecific control products (that is, transfected with ‘double-gene’ expression constructs expressing either a κ light chain and an IgG1 heavy chain or a λ light chain and an IgG1 heavy chain) were also generated to use as comparators.

Several cell lines for each transfection condition were initially evaluated in a non-fed overgrow fermentation to determine IgG titres. These cell lines were selected from an initial panel of ~100 cell lines screened post selection, based on their growth profiles and IgG expression levels determined during the selection process (Supplementary Table 5). No statistical differences were observed for κλ-body, H2-1H1-1, between the overall IgG productivity from the recombinant GS-CHO cell lines transfected with either the triple-gene or the control double-gene expression constructs (Supplementary Fig. 2a). These data suggest that concomitant expression of a κ and λ light chain along with an IgG1 heavy chain in the same cell had no detrimental effect on IgG productivity compared with the expression of either the κ or λ light chain and an IgG1 heavy chain. Total IgG titres from cell lines derived from the transfection with the κλ-body, H2-1H2-2, were slightly higher on average than those derived from the transfection of the κλ-body, H2-1H1-1 (Supplementary Fig. 2a). A fed-batch culture evaluation was performed using a scale-down model on five cell lines from the H2-1H1-1 transfection condition and two cell lines from the H2-1H2-2 transfection condition. At the end of the fed-batch process, titres in excess of 1 g l^−1^ were obtained for all cell lines, confirming the general amenability of the κλ-body bispecific format to the high productivity of a platform mammalian expression system (Supplementary Fig. 2b). Post protein A purification from the harvest culture medium, the material was analysed for the distribution of bispecific κλ-body versus monospecific species. Although distribution of the monospecific species (that is, IgG1κ and IgG1λ) varied between candidates, the κλ-body species was more consistent, ranging between 37% and 46% of the total IgG produced (Supplementary Fig. 2c), suggesting that κλ-body expression is less sensitive to differences in κ and λ light chain levels than the monospecific forms.

The stability of κλ-body expression was confirmed over the course of 50 generations for the recombinant GS-CHO cell lines expressing the H2-1H1-1 κλ-body. For each cell line, the relative bispecific/monospecific levels were determined every ten generations, following a small-scale, non-fed overgrow fermentation culture (Supplementary Fig. 3a). The κλ-body/monospecific species distribution was not found to be altered over the course of the 50 generation study for all 5 cell lines tested (Supplementary Fig. 2d). Furthermore, total IgG and κλ-body titres as well as the time integral of viable cell concentration were found to be stable over time (Supplementary Fig. 3b).

Finally, the robustness of the scaled up high-titre fed-batch platform fermentation process was confirmed at two different manufacturing scales. For κλ-body, H2-1H1-1, a total IgG productivity of 1.5 g l^−1^ was achieved at harvest in a 25 l wave bioreactor fermentation (Supplementary Fig. 2e) with an Ab species distribution of 41%, 12% and 47% for κλ-body monospecific IgG1κ and monospecific IgG1λ, respectively (data not shown). For κλ-body, H2-1H2-2, in a 100 l disposable stirred bioreactor, the IgG productivity reached ~2 g l^−1^ at harvest (Supplementary Fig. 2f), with an Ab species distribution of 45%, 42% and 13% for κλ-body, monospecific κ and monospecific λ IgG1, respectively (Table 2).

**Figure 5:**
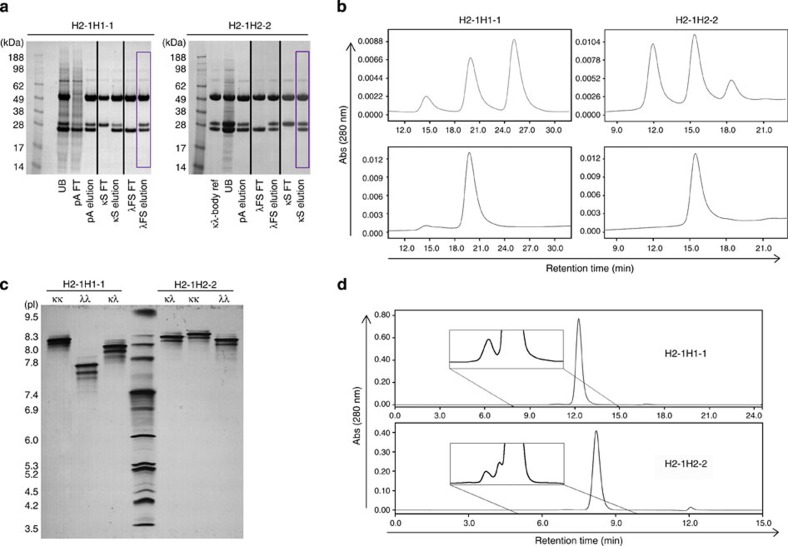
Product quality assessment during the platform purification process. The 25 l wave and 100 l CHO fed-batch cultures expressing H2-1H1-1 and H2-1H1-2, respectively, as depicted in Fig. 5, were clarified to supply loading material for purification. (**a**) Reduced SDS–PAGE analysis of samples taken throughout the platform purification process of the two κλ-bodies H2-1H1-1 and H2-1H2-2. For both, the purification consisted of three consecutive chromatography steps, MabSelect SuRe followed by KappaSelect and LambdaFabSelect (H2-1H1-1), or the two latter steps in the opposite order (H2-1H2-2). Analysed samples were the supernatant unprocessed bulk (UB), the non-retained fraction of the protein A resin (protein A flow through, pA FT), the protein A eluate (pA elution), the KappaSelect flow through and eluate (κS FT and κS elution, respectively), the LambdaFabSelect flow through and eluate (λFS FT and λFS elution, respectively). (**b**) Analysis by HIC-HPLC of the relative abundance of the three IgG forms expressed in the CHO cell culture supernatants (top two panels) and present at the end of the purification process (bottom panels). Samples from the purification process of H2-1H1-1 and H2-1H2-2 are depicted as indicated. The small peak observed with the purified H2-1H1-1 sample does not correspond to a monospecific IgG1κ contamination but to a degradation product, as this peak increases with prolonged incubation at 40 °C (Supplementary Fig. 5). (**c**) Isoelectrofocusing gel analysis of purified κλ-bodies alongside their monospecific controls. (**d**) Determination of aggregate levels in purified κλ-bodies by SEC-HPLC.

The platform three-step ligand-affinity chromatography process (that is, protein A followed by KappaSelect and LambdaFabSelect; Fig. 3a) was evaluated at an industrially relevant scale using culture supernatant from the fed-batch fermentation process. Protein A chromatography conditions previously optimized for fully human recombinant IgG1κ and IgG1λ products expressed using the GS-CHO system were applied. However, as the KappaSelect and LambdaFabSelect chromatography conditions recommended by the supplier were developed for the purification of Fab fragments, we determined optimal capacity, flow-rate and residence time conditions for human IgG1 using an empirical approach (Supplementary Fig. 4). We selected a single set of parameters that suited both chromatography supports, to facilitate downstream processing and allowing the KappaSelect and LambdaFabSelect chromatography steps to be interchangeable. Indeed, as the culture supernatant from the κλ-body, H2-1H1-1, fermentation contained more IgG1λ than IgG1κ monospecific species (Supplementary Fig. 2c), we chose to place the KappaSelect chromatography step ahead of the LambdaFabSelect step, to maximize the loading capacity of the column for κλ-body product. For the κλ-body, H2-1H2-2, where IgG1κ was the predominant monospecific species (Supplementary Fig. 2c), we reversed the two chromatography steps. Table 2 summarizes the overall recoveries obtained for the κλ-body, H2-1H2-2, produced at the 100 l scale for which 66.5 g of purified κλ-body were obtained after downstream processing. The ligand-affinity chromatography steps were found to be robust and selective, with the process yielding a product with the desired product quality attributes (that is, low aggregation and high purity; Fig. 5a–d). Together, these data illustrate the robust platform nature of the scaled-up purification process.

**Figure 6:**
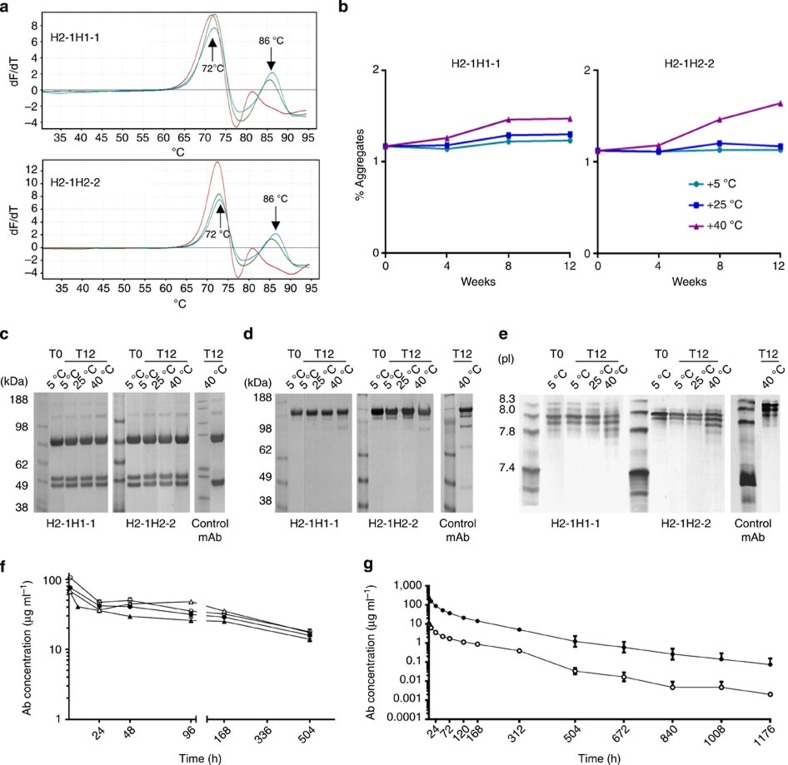
*In-vitro* and *in-vivo* stability profile of κλ-bodies. κλ-bodies H2-1H1-1 and H2-1H2-2 were purified as depicted in Fig. 5, and were assessed for stability *in vitro* and *in vivo*. (**a**) Densitometry scanning fluorimety profiles of κλ-bodies (green) in comparison with the monoclonal controls IgG1κ (blue) and IgG1λ (red). (**b**–**e**) Stability of both purified κλ bodies formulated in 25 mM Histidine, 150 mM NaCl, pH 6 at 10 mg ml^−1^ was determined at 5, 25 and 40 °C for up to 12 (T12) weeks by SEC-HPLC (**b**), reduced SDS–PAGE (**c**), non-reduced SDS–PAGE (**d**) and gel electrofocusing (**e**). (**f**) Serum concentration in mice injected intravenously with 5 mg kg^−1^ of either control mAb or κλ-bodies. Serum titres of H2-1H1-1 (open circles), K15L7-2 (closed triangles), hIgG1κ (open triangles) and hIgG1λ (closed circles) were determined by ELISA. (**g**) Mean (*n*=3) serum concentration ±s.e.m. in cynomolgus monkeys injected intravenously with either 0.5 mg kg^−1^ (open circles) or 10 mg kg^−1^ (closed circles) of K15L7-2.

Next, the stability of the κλ-body format was assessed using H2-1H1-1 and H2-1H2-2 post purification. Thermal stability was investigated using differential scanning fluorimetry (DSF), a technique that assesses domain unfolding over a temperature range. As anticipated due to the fully human and non-engineered nature of the format, the DSF profile of the κλ-body was identical to that of the κ and λ monospecific control Abs (Fig. 6a). Domain unfolding occurred at temperatures comparable to those reported for fully human therapeutic mAbs^28^. Both κλ-bodies were stable, comparable to their κ and λ monospecific control Abs. Furthermore, using a standard formulation (that is, 10 mg ml^−1^ in a standard buffer), their stability was comparable to a standard mAb during storage for 6 months at either 5 °C or 25 °C and 3 months at 40 °C (Fig. 6b–e). In particular, the aggregate levels remained low for 12 weeks at either 5 °C or 25 °C (Fig. 6a). Finally, we assessed the stability of the κλ-body format *in vivo* by evaluating the circulating levels of the H2-1H2-2 and K15L7-2 κλ-bodies over time following a single intravenous administration in mice. Elimination kinetics for the κλ-bodies were comparable to those of control human IgG1κ and IgG1λ (Fig. 6f and Supplementary Table 6). The calculated half lives of the four molecules were between 16 and 20 days, a range in agreement with previous reports for the half life of human IgG1 measured in mice^29^. The production of several candidates including K15L7-2 were scaled up, to perform a single dose, pharmacokinetic study in nonhuman primates (that is, cynomolgous monkeys). The study showed that a single intravenous administration of K15L7-2 (Fig. 6g) and three other candidates (data not shown), at doses of either 0.5 or 10 mg kg^−1^, were well tolerated and the terminal plasma clearance linear.

## Discussion

In natural antibody repertoires, the VH domain is the most diversified region and, in most cases, plays a central role in antigen binding, leading to the general view that the light chain’s contribution is more supplemental than a driver. Despite this observation, we have developed an antibody generation platform that delivered over 100 antibodies sharing a common heavy chain that specifically bind a panel of 10 different proteins. Our results also illustrate the suitability to depend on light-chain diversity for the generation of high-affinity binders as well as to provide potent inhibition for ligand–receptor interactions. Although counterintuitive, our rationale that the light chain can drive specificity and affinity is supported by previous studies using antibody repertoires incorporating limited synthetic diversity, indicating that the CDR L3 can in some cases contribute more to antigen binding than the CDR H3 (ref. 30). Moreover, single-domain antibodies, such as VHH domains found in camelids, as well as several alternative scaffolds rely on two or three loops to establish protein–protein interactions with the target^31^,^32^,^33^. Finally, bovine antibodies often solely rely on their extended CDR H3 for interaction with the antigen, further indicating specificity does not necessarily require multiple interacting loops^34^.

We have shown that diversified light-chain repertoires can be combined with different fixed VH sequences, suggesting that this approach can be generalized. Furthermore, we were able to use either a predefined, fixed VH for a parallel discovery approach (Fig. 1a) or commence by employing the VH of an existing mAb (Fig. 1b). This second scenario can be quite attractive, as it enables the combination of a well-characterized mAb, with potentially suitable properties, into a κλ-body. These two options for VH usage illustrate the flexibility of the technology. In this study, we have successfully used three germlines of the VH3 family. Additional studies using different VH germlines will indicate whether some frameworks are better suited than others for creating κλ-bodies. It is possible that fixing the heavy chain in the libraries could lead to some constraints that might limit the targeting of certain epitopes. Identifying such potential constraints is difficult, as in fact, we generated candidates against ten different targets demonstrating that no major limitation existed at least for this set of antigens. Interestingly, we observed that when the fixed VH used during the selection of the VL was changed for another VH in the IgG, the binding capacity was lost, although the IgG could still be assembled (data not shown), indicating that the heavy chain either directly contributed to the interaction with the antigen or in an indirect manner by interacting with light-chain CDRs. Future structural studies will help to clarify the relative roles of the two chains in fixed heavy-chain antibodies directed against different epitopes.

Most approaches to generate a BiAb aim at forcing the secretion of a unique species of bispecific molecule by the production cell line or rely on the assembly of half antibodies that are secreted independently^11^,^35^. In many formats, the CH3–CH3 interface is modified to favour formation of heavy-chain heterodimers, and swapping of CH1 and CL constant domains or remodelling of the CH1–CL and VH–VL interfaces has been used to promote correct heavy- and light-chain pairing^9^,^12^,^15^. Despite numerous elegant molecular designs, unwanted product-related contaminants, mainly in the form of homodimers, multimers or molecules containing mispaired domains are also produced, and their elimination during downstream processing can be challenging. However, several BiAbs are currently being evaluated in clinical trials, demonstrating that these development hurdles can be overcome^36^,^37^. Here we have taken a different approach by allowing three unmodified immunoglobulin chains to assemble naturally, and using a robust and scalable purification process to retrieve the bispecific κλ-body. The benefit of our strategy is that the κλ-body contains no mutations or nonhuman sequences (except for diversification of the light-chain CDRs that confer target specificity) and is therefore indistinguishable from a human IgG. In line with this, the stability of our κλ-bodies during processing and storage, as well as their pharmacokinetic profiles in mice and non-human primates were comparable to those of a human IgG. Given the limited number of BiAbs having entered clinical trials, the potential impact on immunogenicity of linkers or mutations introduced at the interface between constant domains that are present in different BiAb formats is currently difficult to evaluate. It is however an important point of consideration that will become better understood, as more BiAbs, using different architectures, will enter clinical development for acute and, in particular, chronic indications^38^. It can be anticipated from the observations made during the last decades with therapeutic mAbs, that ‘foreignness’ of sequence, level of aggregates and purity from contaminants for provocation of anti-drug antibodies will also apply to BiAbs^39^,^40^,^41^. The stability and the human nature of κλ-bodies should therefore minimize the risk of immunogenicity. The ‘two-in-one antibody’ is another approach that generates unmodified human IgG capable of binding two different antigens^42^. In this format, the antibody combining site is engineered so that it can bind two targets independently but not simultaneously. This elegant format also benefits from unmodified IgG properties, but because the nature of the antigen bound by each combining site cannot be controlled, this format is not suitable for applications such as T-cell retargeting, where the bridging between two targets is required.

Another important similarity between κλ-bodies and therapeutic mAbs is that the purification process is platform, that is, can be applied to any κλ-body, thus facilitating parallel development of multiple candidates. By establishing stable cell lines using a recombinant CHO system and scaling up the bioprocess to 100 l, we demonstrated compatibility with industrial scale manufacturing. Notably, the κλ-bodies are also able to perform BiAb mechanisms by, for example, mediating co-engagement of two antigens in *cis* at surface of the same cell or in *trans*, for example, bridging two cells.

In summary, the κλ-body represents a unique human BiAb format that exploits the antibody light chain for antigen-binding specificity and robust downstream processing. The stability and fully human nature of the κλ-body should be particularly well suited for therapeutic intervention, especially in chronic settings.

## Methods

### Fixed VH library construction

Human VH and VL germlines were synthesized according to the nucleotide sequence found in IMGT^17^. Each of the fixed VH was first cloned into the phagemid vector pNDS1 (ref. 43) using NcoI/XhoI restriction sites. The CDR1 and CDR2 of the VL genes were diversified using degenerate oligonucleotides using DMT, RVK and NHT codons depending on the number of randomized positions. Diversified CDR L3 sequences (NNS, NHT and RVK codons) were then introduced by a Type IIS restriction cloning approach^43^. After ligation, recombinant pNDS1 was electroporated into *Escherichia coli* TG1 cells. Some libraries were also generated by cloning naturally occurring VL from circulating B cells isolated from 50 healthy human donors. The different libraries that were generated are described in Supplementary Table I.

### Recombinant protein production

All antigens were produced in mammalian cells and included an Avi-Tag-mediating single-site biotinylation, to facilitate immobilization for phage display selection as previously described^44^.

### Library rescue and phage display selections

TG1 cells were grown at 37 °C (240 r.p.m.) in 2 × TYAG (100 μg per ml ampicillin, 2% glucose) medium. At OD600=0.4–0.5, the libraries were rescued by superinfection with M13KO7 helper phage for 1 h at 37 °C (100 r.p.m.). Culture medium was then replaced with 2 × TYAK (100 μg per ml ampicillin and 50 μg per ml kanamycin) and TG1 were grown o/n at 30 °C (280 r.p.m.). Phage were purified and concentrated from the culture supernatant by two precipitations with one-third v/v of 20% PEG-8000/2.5 M NaCl (Sigma) and resuspended in TE buffer, followed by ultracentrifugation on a CsCl gradient. The band corresponding to the purified phage particles was recovered, dialysed against TE buffer and titrated by infecting TG1 cells.

Aliquots from each scFv phage library (10^12^ pfu) used in the study was blocked with PBS containing 3% (weight per volume) skimmed milk and then deselected either on streptavidin (Dynal) or neutravidin (ThermoFisher) magnetic beads, or on target-negative cells. Recombinant protein was biotinylated, coated on magnetic streptavidin beads and incubated with the deselected phage for 2 h (room temperature (RT)). For cell surface targets, 1–10 × 10^7^ cells naturally expressing or transfecetd with the surface antigen were used. Nonspecific phage were eliminated by five washes with PBS/0.1% Tween 20 and two washes with PBS. For selections on cells, only PBS was used for the washing steps. Bound phage were eluted with 10 mM triethylamine TEA (Sigma) or 76 mM citric acid, and then neutralized with 1 M Tris-HCl, pH 7.4 (Sigma). The eluate was added to 10 ml of exponentially growing *E. coli* TG1 cells and incubated for 1 h at 37 °C (100 r.p.m.). An aliquot of the infected TG1 was serial diluted to titre the selection output. The remaining infected TG1 were spread on 2 × TYAG agar Bioassay plates. After overnight incubation at 30 °C, bacteria were scraped off with 2 × TY medium and aliquots were stored at −80 °C in 17% glycerol. For subsequent rounds of selection, bacterial culture supernatant containing 10^10^ pfu were used.

### scFv screening

After three to five rounds of phage-display selection, individual clones were picked and grown in 2 × TYAG medium in 96-well deepwell plates. ScFv expression was induced by isopropyl-β-D-thiogalactoside addition (0.02 mM, final concentration) overnight at 30 °C (250 r.p.m.). Cells were centrifuged and the pellet was re-suspended in150 μl TES buffer (50 mM Tris/HCl, pH 8, 1 mM EDTA, pH 8, 20% sucrose, complemented with complete protease inhibitor, Roche). A hypotonic shock was produced by adding 150 μl of diluted TES buffer (1/5 TES in water) followed by incubation on ice for 30 min. Plates were then centrifuged (3,000*g*, 5 min) and supernatants were used for screening by ELISA using recombinant proteins or for cell surface binding, using an FMAT8200 reader (Applied Biosystems). For ELISA, 96-well streptavidin-coated plates (Greiner) were incubated for 30 min (RT), with 1 μg per well of biotinylated recombinant proteins. After washing the assay plates three times with PBS/0.05% Tween 20, 50 μl of the periplasmic supernatants containing scFv were transferred to the wells and incubated for 2 h at RT. Binding scFv were detected with mouse anti-cmyc (1 μg ml^−1^; Invitrogen 13–2500) and anti-mouse IgG Fcγ-HRP (dilution 1:5,000; Jackson 109-035-098) antibodies. The assay was developed with TMB substrate (Sigma), the reaction stopped with H_2_SO_4_ 2N and the absorbance read at 450 nm. For cell surface binding, cells expressing the target at their surface and target-negative cells were resuspended in PBS/2% BSA/0.1% NaN_3_ and distributed in 384-well clear-bottom plates (Corning) at 3,000 cells per well. Seventy microlitres of mixed periplasmic preparation and Penta-His AlexaFluor 647 conjugate (dilution 1:2,000; Qiagen 35370) were added to the cells. The assay plates were incubated for 3 h at RT and binding scFv were detected with the FMAT8200 reader (Applied Biosystems).

### Cell lines

All cell lines were obtained from ATCC and were tested for mycoplasma contamination (Jurkat TIB-152; Raji CCL-86; MCF-7 HTB-22; A431 CRL-1555; DS-1 CRL-11102). A stable CHO cell line expressing human CD47 was established by selection in puromycin-containing media of CHO cells transfected by electroporation with a pEAK8 vector (Edge Bio) in which the full-length coding sequence of human CD47 was cloned (GenBank X69398).

### Bio-layer interferometry

The Octet RED96 (Fortebio, Pall) was used to measure affinity and kinetic parameters of several mAbs for their target. Biosensor tips and kinetic buffer (KB: 1 mM phosphate, 15 mM sodium chloride, 0.002% Tween 20, 0.005% sodium azide, 0.1 mg per ml BSA, pH 7.4) were purchased from Pall (Basel, Switzerland). Black 96-well microplates were obtained from Greiner Bio One. All experiments were performed at 30 °C.

For hCD47 and hCD19, anti-human Fc capture biosensor tips were first hydrated in KB buffer for 10 min. Next, as recommended by the manufacturer, tips were preconditionned by applying a regeneration cycle in 10 mM glycin, pH 1.7. After a baseline step in KB for 2 min, mAbs were captured at 5 μg ml^−1^ for 15 min and 1,000 r.p.m. A second baseline step for 2 min allowed for signal stabilization after mAb capture. The association and dissociation steps were monitored for 7 min and a final regeneration cycle was performed to strip biosensor tips for following experiments. Kinetics were obtained with serial dilutions of proteins on seven concentrations, with a dilution factor of 2. For hCD47, a starting concentration of 100 nM was used for all kinetics. Concerning hCD19, a starting concentration of 706 nM was applied for IgGL7-1 and IgGL7-2, whereas a higher starting concentration of 3 μM was used for IgG1B7. A 1:1 global fitting model was used to analyse data and obtain association and dissociation rates, and the *K*_D_.

For hEpCAM, streptavidin biosensor tips were used due to very low signals obtained with the anti-human Fc capture capture format. Loading experiments were performed to evaluate an appropriate loading concentration of 2 μg ml^−1^, which gives a sufficient binding signal with a limited avidity effect. Biosensor tips were hydrated in KB for 10 min, a baseline in KB was applied for 2 min and then *in-vivo* biotinylated hEpCAM at 2 μg ml^−1^ in KB was captured on the surface. A 1.3-nm capture signal was reached after a 10-min incubation. After a second baseline for 2 min in KB, biosensor tips were dipped into wells containing mAbs at concentrations ranging from 50 to 0.78 nM, with a twofold serial dilution in KB. Association and dissociation steps were monitored for 10 min. A regeneration scouting was performed to identify the best regeneration solution, which allows a complete elimination of bound antibodies without affecting the tip-binding capacity. Glycin (10 mM, pH 1.7) was used with three regeneration/neutralization cycles. Data were analysed using a 1:1 global fitting model.

### Small-scale BiAb production and purification

Transformed Human Embryo Kidney monolayer epithelial cells (PEAK cells; Edge Bio) were maintained in 5% CO_2_ at 37 °C in a humidified atmosphere in DMEM (Invitrogen) containing 10% FCS (Sigma-Aldrich, St Louis, MO) and supplemented with 2 mM glutamine (Sigma-Aldrich); this medium is referred to as complete DMEM.

Transient transfections were performed using a mix containing 30 μg of DNA and 42 μl of Lipofectamine 2000 transfection reagent (Invitrogen) in 1.4 ml of DMEM for 10^7^ cells per T175 flask. Antibody concentration in the serum-containing supernatant of transfected cells was measured at several time points during the production using the Bio-layer interferometry technology. An OctetRED96 instrument and Protein A biosensors were used for quantitation (Pall). Briefly, 200 μl of supernatant were used to determine IgG concentration; biosensors were preconditioned and regenerated using 10 mM glycine, pH 1.7, solution and IgG calibrators diluted in conditioned PEAK cell medium were prepared for standard curve generation. Concentrations were determined using the dose–response 5PL-weighted Y standard curve equation and an initial slope-binding rate equation. According to antibody concentration, supernatants were harvested 7 to 10 days after transfection and clarified by centrifugation at 1,300*g* for 10 min. The purification process was composed of three affinity steps. First, the CaptureSelect IgG-CH1 affinity matrix (Life Technologies) was washed with PBS and then added to the clarified supernatant. After incubation overnight at 4 °C, supernatants were centrifuged at 1,000 *g* for 10 min, the supernatant was discarded and the resin washed twice with PBS. Then, the resin was transferred to spin columns and a solution containing 50 mM glycine at pH 2.7 was used for elution. Several elution fractions were generated, pooled and desalted against PBS using 50 kDa Amicon Ultra Centrifugal filter units (Merck KGaA). The final product, containing total human IgG from the supernatant, was quantified using a Nanodrop spectrophotometer (NanoDrop Technologies, Wilmington, DE) and incubated for 15 min at RT and 20 r.p.m. with the appropriate volume of KappaSelect affinity resin (GE Healthcare). Incubation, resin recovery, elution and desalting steps were performed as described previously. The last affinity-purification step was performed using the LambdaFabSelect affinity resin (GE Healthcare), applying the same process as for the two previous purifications. The final product was quantified using a Nanodrop.

### Quantification of cell surface receptor density

CD47 and CD19 expression was determined using QFIKIT (Dako K0078) containing five different populations of beads bearing different numbers of mouse mAb. Cells were labelled with a saturating dose (10 μg ml^−1^) of primary mouse mAb against the antigen of interest (anti-CD19, R&Dsystems MAB4867; anti-CD47, eBiosciences B6H12). Beads and cells were then stained with an anti-mouse Fc-FITC (dilution 1:50; included in the Dako kit) and analysed by flow cytometry. Cell fluorescence is then compared with the bead standards to estimate antigen quantity on the surface of cells.

### Retargeting of human T cells by BiAbs

All cell lines were obtained from ATCC. Peripheral blood mononuclear cells were isolated from buffy coats and T cells were purified by a negative selection using a human T-cell enrichment kit (R&D systems). Freshly isolated T cells were maintained for 4 days in precoated flask with OKT3 (10 μg ml^−1^; Abcam ab86883) and anti CD28 (10 μg ml^−1^; Abcam ab25234) in complete RPMI 1640 medium supplemented with 30 U ml^−1^ of h-IL2 (R&D systems). The day before the test, T cells were transferred in an uncoated flask. The purity of activated T cells was confirmed by FACS analysis. MCF-7 (EpCAM-positive) were treated with serial dilutions of CD3 × EpCAM BiAb or control antibodies together with activated T cells (E:T=5:1) overnight at 37 °C in a round-bottom 96-well plate. Released lactate dehydrogenase was detected with a cytotoxicity detection kit (Roche). Data were processed and percentages of specific lysis were calculated and analysed using GraphPad Prism6 software.

### CD47/SIRPα-binding assay

The SIRPα-CD47-binding and inhibition assay was performed as follows: Raji or DS-1 cells were washed once in FMAT buffer (PBS, 2% BSA, 0.1% NaN_3_) and incubated for 30 min at RT in FMAT Buffer supplemented with 10% mouse serum to block Fc receptors. Cells were washed and incubated with or without different concentrations of the test antibody (mAb or biAb) for 50 min at RT. In parallel, hexahistidine-tagged soluble hSIRPα (200 ng ml^−1^), pentaHIS-biotin antibody (200 ng ml^−1^; Qiagen 34440) and Streptavidin-Cy5 (Invitrogen SA1011; dilution 1:5,000) were mixed and incubated for 50 min. Thirty microlitres of the mixture and 70 μl cell suspension (containing 3,000 cells) were added to each well of a 384-well clear-bottom plate (Corning) and incubated for 3 h. SIRPα binding to CD47 was determined using an FMAT8200 reader (Applied Biosystems).

### Mesothelin/MUC16-blocking assay

The Mesothelin-MUC16-blocking assay was performed as follows: CHO cells transfected with human Mesothelin were incubated with or without different concentrations of the test antibody (mAb) for 30 min at RT in FMAT buffer (PBS, 2% BSA, 0.1% NaN_3_). In parallel, hexahistidine-tagged soluble human MUC16 (50 ng ml^−1^; R&D system) and pentaHIS-AlexaFluor 647 antibody (dilution 1:400; Qiagen) were mixed and incubated for 30 min. Twenty microlitres of the mixture and 80 μl of cell suspension (containing 3,000 cells) were added to each well of a 384-well clear-bottom plate (Corning) and incubated for 2 h. MUC16 binding to Mesothelin was determined using an FMAT8200 reader (Applied Biosystems).

### Stable cell line generation and large-scale production

Glutamine synthetase (GS) expression vectors and the CHOK1SV cell line were provided by Lonza Biologics. Cells were transfected with linear DNA and selected for stable genome integration by culture in the absence of exogenous L-glutamine and in the presence of 50 μM L-methionine-DL-sulphoximine. For small-scale (50 ml Erlenmeyer flask) non-batch fermentations, cells were inoculated at 2 × 10^5^ cells per ml in CD CHO medium (Invitrogen) and harvested following 10 days of culture. For small-scale (50–100 ml Erlenmeyer flask), 25 l WAVE Bioreactor (GE Healthcare) and 100 l STR 200 fed-batch fermentations, the Lonza Biologics media and feed version 7.0 platform process was followed. Cultures were harvested at day 15 post-inoculum or within 24 h of the viability dropping below 70%, whichever came first.

Culture supernatant at harvest was clarified by depth filtration using a D0HC/B1HC/filtration train (Millipore) followed by a 0.22-μm filtration into a flexboy (Sartorius) and subjected to three-step ligand affinity chromatography comprising the MabSelectSure, KappaSelect and LambdaFabSelect affinity resins (GE Healthcare). Running conditions are as described in figure legends. KappaSelect and LambdaFabSelect affinity resins were loaded at pH 7.0. To remove unbound proteins, all resins were washed sequentially with 50 mM sodium phosphate, 250 mM NaCl, pH 7.0, and 50 mM sodium phosphate, 1 M NaCl, pH 7.0. Bound proteins were eluted with 50 mM glycine/HCl, pH 3.5. Following purification, the κλ-body product was formulated into 25 mM histidine, 125 mM NaCl, pH 6.0, for subsequent analyses. For the assessment of stability during storage, κλ-body was aliquoted into type I glass vials sealed with flurotec-coated rubber stoppers (West Pharmaceutical).

### Bispecific characterization

Purified biAb were analysed by electrophoresis in denaturing and reducing conditions. The Agilent 2100 Bioanalyzer was used with the Protein 80 kit as described by the manufacturer (Agilent Technologies). Four microlitres of purified samples were mixed with sample buffer supplemented with dithiothreitol (Sigma-Aldrich). Samples were heated at 95 °C for 5 min and then loaded onto the chip.

BiAb distribution (in a mix with monospecific species) and integrity was assessed by isoelectric focusing (Cambrex pH 3–10 IsoGel agarose plates) and SDS–PAGE analysis (Novex NuPAGE 4–12% Bis Tris gels). Distribution and purity was quantified by HIC-HPLC with a ProPac HIC-10 column (Dionex) using 10 mM sodium phosphate, 1.0 M ammonium sulfate, pH 3.5, mobile phase and a 10 mM sodium phosphate, 10 % acetonitrile, pH 3.5, buffer for gradient elution. The limit of detection of this HIC-HPLC method is 1%. Aggregate and fragment levels were determined by SEC-HPLC with a Biosep-SEC-s3000 column (Phenomenex) for H2-1H1-1 or a TSKgel G3000SWXL column (Tosoh) for H2-1H2-2. Both columns were run using a 200-mM sodium phosphate, pH 7.0, mobile phase. DSF was performed with the SYPRO Orange fluorescent probe (Invitrogen) using a Rotor-Gene Q 2plex HRM platform (Qiagen). Five micrograms of antibodies formulated in PBS were mixed with SYPRO Orange (Invitrogen) diluted from a 20 × concentrate in a total volume of 20 μl. Samples in 0.2-ml PCR tubes (Qiagen) were loaded onto 72-well rotor disc, which was mounted on a Rotor-Gene Q 2 plex HRM cycler (Qiagen). Fluorescence was then measured with the HRM channel and plotted against temperature using the following parameters: temperature ramp from 35 to 95 °C, 1 °C rise of temperature at each step, 5 s between each step.

The melting temperature was determined with the Rotor-Gene Q software (Qiagen, version 2.1.0) and defined as the turning point of the sigmoidal-like curve of fluorescence plotted against temperature. The melting temperature was calculated by assigning the minimum of the corresponding inverted second derivative curve. All samples were tested for endotoxin contamination according to the Limulus Amebocyte Lysate test (Charles River Laboratories).

### Pharmacokinetics

All experiments were performed with approval from the Geneva cantonal veterinary office for animal experimentation. The single dose pharmacokinetic profile of the BiAb in mice was determined by injecting 5 mg per kg in the tail vein of 8-week-old female C57BL/6J mice. Serum human IgG concentrations were determined using the FastELYSA system (RD-Biotech). Serum from three animals was tested for each time point. BiAb concentrations was also measured using a sandwich ELISA format comprising a biotin-coupled anti-λ domain capture reagent and an horeseradish peroxidase-coupled anti-κ mAb detection reagent (Jackson ImmunoResearch), to confirm the integrity of the κλ-body. An *in-vivo* study was performed in non-human primates, to determine the pharmacokinetic profile of the κλ-body K15L7-2. Female cynomolgus monkeys (three per group) of 2.5–3.7 kg and in the age range of 2–3 years were dosed once by intravenous administration of K15L7-2 at 0.5 or 10 mg kg^−1^. The animals were inspected twice daily and any clinical observations were recorded. Blood samples were collected at various time points for 8 weeks. The serum concentrations of K15L7-2 were determined by ELISA, using a generic human IgG detection immunoassay. This study was performed at Covance Laboratories GmbH test facility in Munster. All procedures in the study were in compliance with the German Animal Welfare Act and were approved by the local Institutional Animal Care and Use Committee.

## Author contributions

N. Fischer, G.E., K.M., W.F. and M.K.-V. conceived and designed the research, analysed the data and composed the manuscript. G.M., E.D., A.L., F.G., U.R., V.M., P.M., F.R., S.C., S.F., L.B., G.D., N.B. and N.C. carried out the experiments regarding library building, antibody discovery, bispecific generation and *in-vitro* characterization experiments. They analysed the data and generated figures for the manuscript. N. Fouque., J.-F.D., S.R., L.D.G., Y.P., P.-A.C., M.A., G.P., M.C. and D.S carried out stable cell line generation, large-scale BiAb expression, purification, as well as all analytical work. They analysed the data and generated figures for the manuscript. E.D., V.M., L.C., V.B. and Z.J. carried out *in-vivo* experiments, pharmacokinetic analysis and generated figures for the manuscript. All authors discussed and commented on the manuscript.

## Additional information

**How to cite this article:** Fischer, N. *et al.* Exploiting light chains for the scalable generation and platform purification of native human bispecific IgG. *Nat. Commun.* 6:6113 doi: 10.1038/ncomms7113 (2015).

## Supplementary Material

Supplementary Information Supplementary Figures 1-5 and Supplementary Tables 1-6

## Figures and Tables

**Table 1:** Libraries used for phage display selections and outcome of screening against a panel of human proteins

**Target**	**Library kappa**	**Library lambda**	**scFv** *	**IgG**†	**VL germline diversity**‡
**hCD47**	23KN, 23K3, 23K123, S23K	23LN, 23L3, 23L13, 23L123, S23L	85/380	15/20	K1-6, K1-9, K1-33, K1-39, K4-1 L1-40, L1-44, L1-47, L1-51, L2-14, L3-1, L6-57
	S48K	S48L	10/23	ND	K1-33, K1-39
**hEpCAM**	23KN, 23K13, 23K123, 48K	23LN, 23L3, 23L13, 23L123	60/296	13/15	K1-5, K1-12, K1-33, K1-39, K3-11, K3-15, K3-20 L1-40, L1-44, L1-47, L1-51, L3-21, L6-57
**hEGFR**	23K13, 23K123	23LN, 23L3, 23L13, 23L123	17/42	6/6	K1-33 L1-40, L1-44, L1-51, L6-57
	S48K	S48L	1/7	ND	L2-14
	S30K	S30L	1/7	ND	K1-33
**hHER2**	23K13	23LN, 23L3, 23L13, 23L123	20/41	4/6	K1-33, K1-39, K3-15 L1-40, L1-51, L2-14, L6-57
**hCD19**	23KN, 23K3, 23K13, 23K123,	23LN, 23L3, 23L13, 23L123	176/986	6/85	K1-5, K1-13, K1-33, K1-39, K3-11, K3-15, K3-20, K4-1 L1-40, L1-44, L1-51, L2-14, L3-1, L6-57
**hIFNγ**	23K3	23L3	5/10	3/4	L6-57
**hIL6R**	23K3	23L3	15/21	9/9	K3-11 L2-14
	S30K	S30L	12/16	ND	K1-33, K3-11 L2-14
**hFOLR1**	23KN, 23K3, 23K13, 23K123	23LN, 23L3, 23L13, 23L123	53/177	18/18	K1-5, K1-12, K1-39 L1-40, L1-44, L2-14, L2-23, L3-21, L6-57
**hMSLN**	23KN, 23K3, 23K13, 23K123	23LN, 23L3, 23L13, 23L123	95/189	20/30	K1-5, K1-9, K1-33, K1-39, K2D-29, K3-11, K3-15, K3-20 L1-40, L1-44, L1-47, L2-14, L3-21, L6-57
**hGPC3**	23KN, 23K3, 23K13, 23K123	23LN, 23L3, 23L13, 23L123	131/213	9/16	K1-5, K1-9, K1-33, K1-39, K2D-29, K3-11, K4-1 L1-40, L1-44, L1-47, L1-51, L2-11, L2-14, L3-21, L6-57

ND, not determined; scFv, single-chain Fv; VH, variable heavy chain; VL, variable light chain.

The different fixed VH libraries combined with κ or λ light chains are described in Supplementary Table S1.

^*^The number of unique scFv sequences identified as well as the total number of sequenced candidates are indicated.

^†^The total number of scFv reformatted into IgG and the number IgG that could be confirmed as specific for the target are reported.

^‡^The identity of different VL germlines found in the positive scFvs is given based on the IMGT nomenclature^17^.

**Table 2:** In-process sample analysis (HIC-HPLC) of in-process purification samples for H2-1H2-2 at 100 l scale.

**Purification step**	**% κκ mono**	**% λλ mono**	**% κλ body**	**% IgG (step/overall)**	**% κλ-body (step/overall)**
MabSelect SuRe	42	13	45	93/93	93/93
LambdaFabSelect	0	11	89	47/43	89/83
KappaSelect	0	0	100	81/35	90/74
