# Supplementary material for: Exploiting light chains for the scalable generation and platform purification of native human bispecific IgG
Source: Nat Commun. 2015 Feb 12;6:6113. doi: 10.1038/ncomms7113 (PMC4339886; doi:10.1038/ncomms7113)
Supplement: Supplementary Information — Supplementary Figures 1-5 and Supplementary Tables 1-6 [file ncomms7113-s1.pdf]

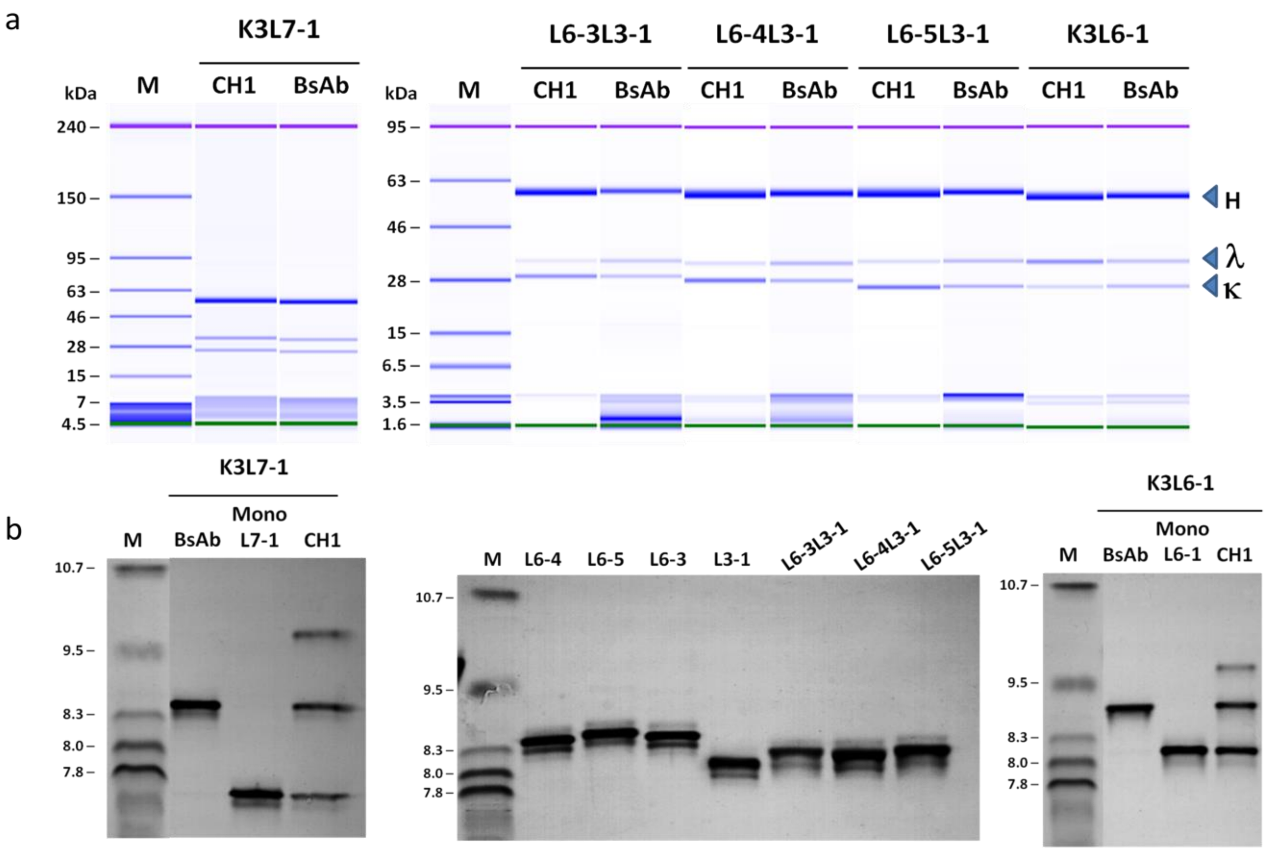

Supplementary Figure 1. Characterization of κλ-bodies. (**a**) Electrophoresis analysis on an Agilent Bioanalyzer 2100 of total IgG obtained after the first IgG-CH1 Capture Select affinity chromatography step and after the third affinity chromatography steps for several κλ-bodies. The bands corresponding to the common heavy chain and the κ and λ light chains are indicated. (**b**) Isoelectric focusing gels of purified κλ-bodies, mAbs or fraction obtained after IgG-CH1 Capture Select affinity chromatography.

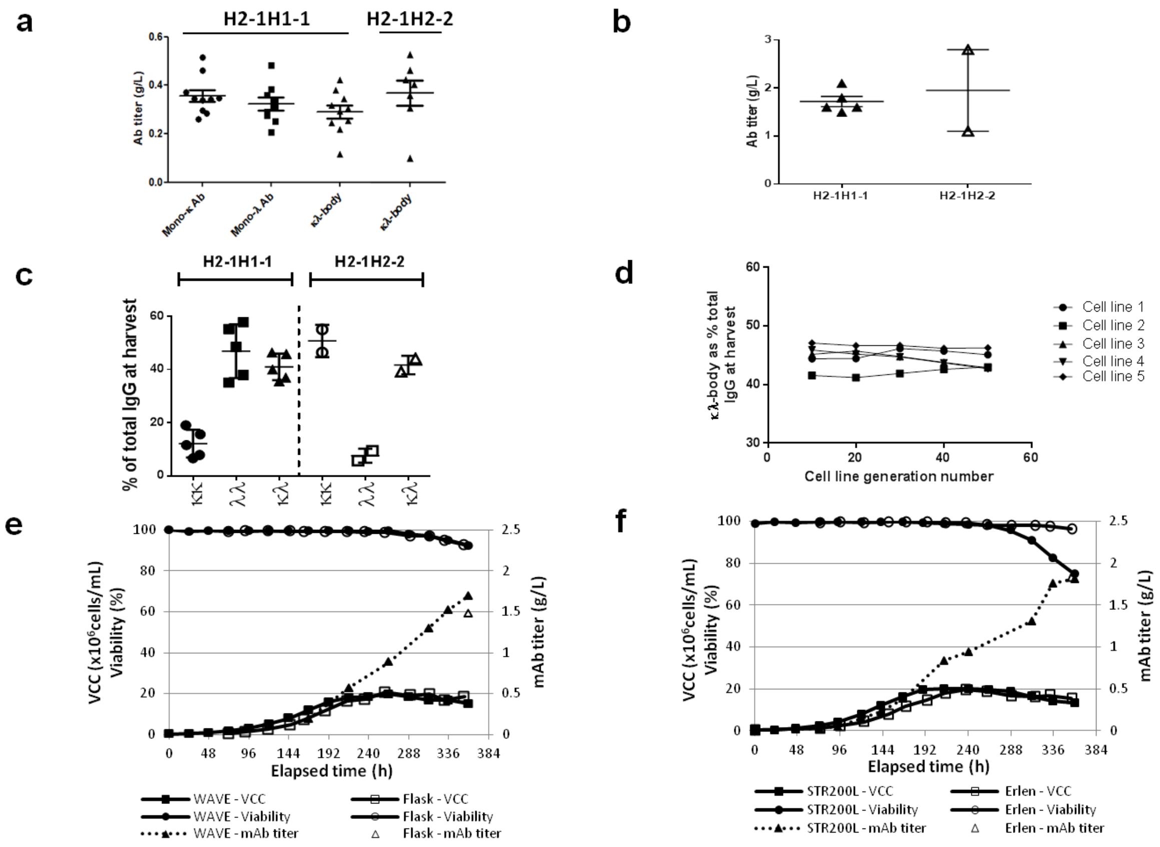

Supplementary Figure 2. Demonstration of high titer κλ-body small and large scale expression. (**a**) Total IgG titer obtained at harvest of 50 mL batch cultures of stable CHO cell lines transfected with either triple gene vectors (i.e., κλ-bodies H2-1H1-1 and H2-1H2-2), or one of their corresponding double gene vector (i.e., mAbs H2-1 and H1-1). (**b**) Total IgG titers reached for the κλ-bodies H2-1H1-1 and H2-1H2-2 at harvest of 50 mL cultures of top stable CHO cell lines operated in fed-batch mode. (**c**) Distribution of the three IgG species in the supernatant of stable CHO cell lines transfected with triple gene vector and expressing κλ-bodies H2-1H1-1 and H2-1H2-2. (**d**) Assessment of the stability of expression of a panel of five CHO cell lines expressing the κλ-body H2-1H1-1 over 50 generations. (**e**) Scale up to 25 L in wave cultures in fed-batch of stable CHO cell line expressing the κλ-body H2-1H1-1. (**f**) Scale up to 100 L in a disposable stirred bioreactor with cell line expressing κλ-body H2-1H2-2.

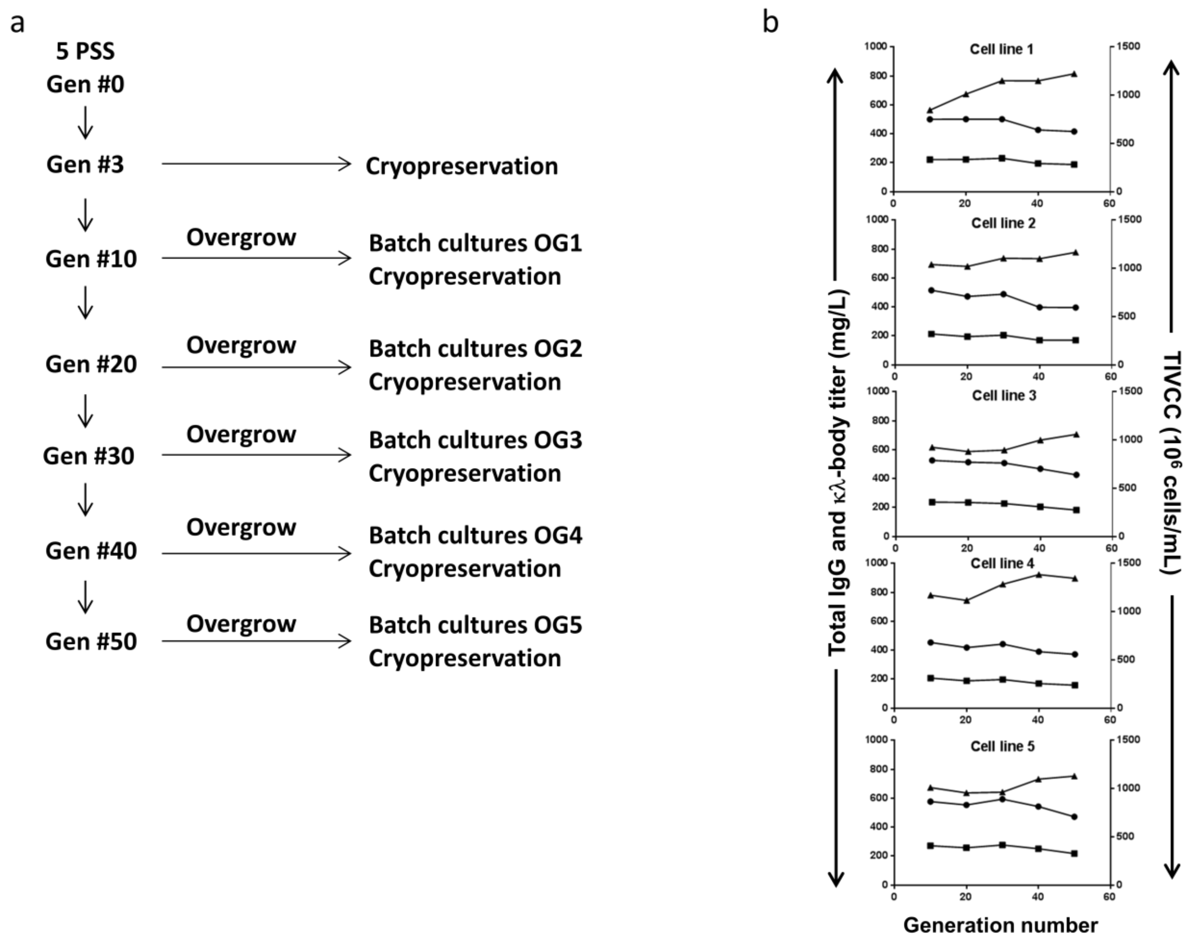

Supplementary Figure 3. Assessment of stability of 5 CHO cell lines expressing κλ-body H2-1H1-1 for 50 generations. (a) Flow diagram of the cell line stability study. (b) Total IgG (circles), κλ-body (squares) titers and time integral of viable cell concentration (TIVCC, triangles) at harvest of batch overgrown cultures. Abbreviations: Gen #: generation number; OG: overgrown.

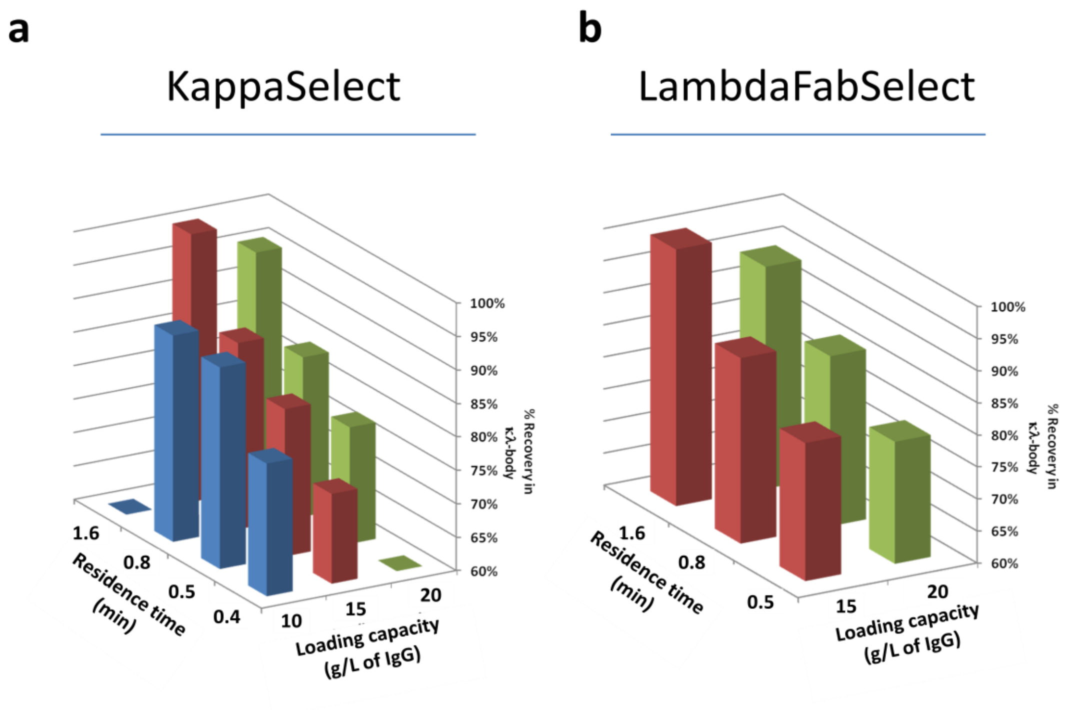

Supplementary Figure 4. Optimization of chromatography conditions with KappaSelect and LambdaFabSelect. Percentage recovery of κλ-body obtained during KappaSelect (a) and LambdaFabSelect (b) chromatography when varying loading capacities and residence times. Purification runs were performed with 15.7 mL resin packed columns (1 cm diameter, 20 cm height).

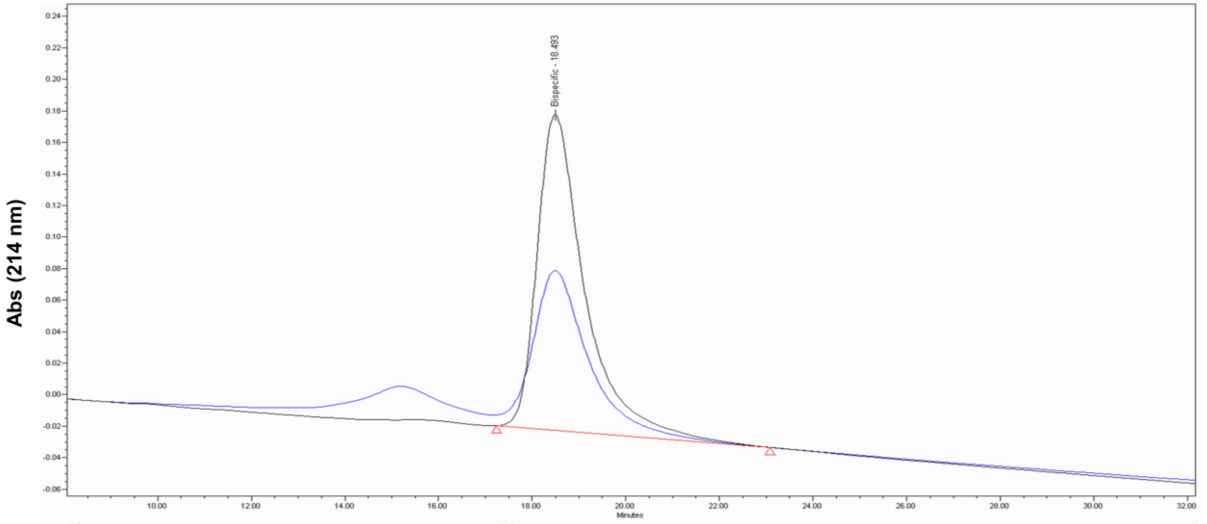

T0; H2-1H1-1

T12 Weeks; 40°C; H2-1H1-1

H2-1H1-1	% variant (Rt=15min)
T0	<LOQ
T12 40°C	22.4%

Supplementary Figure 5. Degradation product monitored by HIC-HPLC. Samples of the κλ-body H2-1H1-1 at T0 and after 12 weeks incubation at 40°C were analyzed by HIC-HPLC. The relative abundance of the degradation product is indicated.

Supplementary Table 1. Fixed VH antibody libraries. The fixed VH sequence, the different VL germlines integrated in each library as well as the CDR diversification strategy that was applied are indicated. Naturally occurring rearranged VL genes were isolated from circulating B cells and cloned in libraries as indicated (*). The library sizes were determined by titration of transformed TG1 cells. The number of sequences in the correct reading frame was determined by sequencing of 50 to 150 transformants per library.

			Position (IMGT): codon randomization				
**Name**	**Fixed VH**	**VL germlines**	**CDRL1**	**CDRL2**	**CDRL3**	**Size**	**In frame sequences**
**23K3**	HV3-23-A	IGKV1-33; IGKV1-39; IGKV3-11; IGKV3-15; IGKV3-20; IGKV4-1	Germ.	Germ.	107-113: NNS<br>107-113; 115: NNS<br>107-114; 116: NNS	6.6E+09	95%
**23K13**	HV3-23-A	IGKV1-33; IGKV1-39; IGKV3-15	28; 30; 31; 32: RVK/DMT	Germ.	107-113: NNS<br>107-113; 115: NNS<br>107-114; 116: NNS<br>107-113: NHT/RVK<br>107-109; 114-115: NHT/RVK<br>107-117: NHT/RVK	1.4E+10	90%
		IGKV3-20	28; 30; 31; 32; 33: RVK/DMT	Germ.	107-113: NNS<br>107-113; 115: NNS<br>107-114; 116: NNS<br>107-113: NHT/RVK<br>107-109; 114-115: NHT/RVK<br>107-117: NHT/RVK		
		IGKV4-1	31 to 38: RVK/DMT	Germ.	107-113: NNS<br>107-113; 115: NNS<br>107-114; 116: NNS<br>107-113: NHT/RVK<br>107-109; 114-115: NHT/RVK<br>107-117: NHT/RVK		

			Position (IMGT): codon randomization				
**Name**	**Fixed VH**	**VL germlines**	**CDRL1**	**CDRL2**	**CDRL3**	**Size**	**In frame sequences**
**S23K**	HV3-23-B	IGKV1-33; IGKV1-39; IGKV3-11; IGKV3-15; IGKV3-20; IGKV4-1	Germ.	Germ.	107-113: NNS<br>107-113; 115: NNS<br>107-114; 116: NNS	3.4E+08	88%
**S30K**	HV3-30-B	IGKV1-33; IGKV1-39; IGKV3-11; IGKV3-15; IGKV3-20; IGKV4-1	Germ.	Germ.	107-113: NNS<br>107-113; 115: NNS<br>107-114; 116: NNS	3.1E+08	87%
**S48K**	HV3-48-B	IGKV1-33; IGKV1-39; IGKV3-11; IGKV3-15; IGKV3-20; IGKV4-1	Germ.	Germ.	107-113: NNS<br>107-113; 115: NNS<br>107-114; 116: NNS	2.8E+08	92%
**48K**	L3-1_HV3-48	Naturally occurring*	Naturally occurring*	Naturally occurring*	Naturally occurring*	9.5E+09	94%
		IGKV1-33; IGKV1-39; IGKV3-11; IGKV3-15; IGKV3-20; IGKV4-1	Germ.	Germ.	107-113: NNS<br>107-113; 115: NNS<br>107-114; 116: NNS		
		IGKV1-33; IGKV1-39; IGKV3-11; IGKV3-15	30; 31: RVK/DMT	56; 66**: RVK/DMT	107-113: NNS<br>107-113; 115: NNS<br>107-114; 116: NNS<br>107-113: NHT/RVK<br>107-109; 114-115: NHT/RVK<br>107-117: NHT/RVK		
			28; 30; 31; 32: RVK/DMT	Germ.	107-113: NNS<br>107-113; 115: NNS<br>107-114; 116: NNS<br>107-113: NHT/RVK<br>107-109; 114-115: NHT/RVK<br>107-117: NHT/RVK		

			Position (IMGT): codon randomization				
**Name**	**Fixed VH**	**VL germlines**	**CDRL1**	**CDRL2**	**CDRL3**	**Size**	**In frame sequences**
**48K**	L3-1_HV3-48	IGKV3-20	30; 31; 32: RVK/DMT	56; 66**: RVK/DMT	107-113: NNS<br>107-113; 115: NNS<br>107-114; 116: NNS<br>107-113: NHT/RVK<br>107-109; 114-115: NHT/RVK<br>107-117: NHT/RVK	9.5E+09	94%
			28; 30; 31; 32; 33: RVK/DMT	Germ.	107-113: NNS<br>107-113; 115: NNS<br>107-114; 116: NNS<br>107-113: NHT/RVK<br>107-109; 114-115: NHT/RVK<br>107-117: NHT/RVK		
		IGKV4-1	32; 33; 35: RVK/DMT	56; 66**: RVK/DMT	107-113: NNS<br>107-113; 115: NNS<br>107-114; 116: NNS<br>107-113: NHT/RVK<br>107-109; 114-115: NHT/RVK<br>107-117: NHT/RVK		
			31 to 38: RVK/DMT	Germ.	107-113: NNS<br>107-113; 115: NNS<br>107-114; 116: NNS<br>107-113: NHT/RVK<br>107-109; 114-115: NHT/RVK<br>107-117: NHT/RVK		

			Position (IMGT): codon randomization				
**Name**	**Fixed VH**	**VL germlines**	**CDRL1**	**CDRL2**	**CDRL3**	**Size**	**In frame sequences**
**23K123**	HV3-23-A	IGKV1-33; IGKV1-39; IGKV3-11; IGKV3-15	30; 31: RVK/DMT	56; 66**: RVK/DMT	107-113: NNS<br>107-113; 115: NNS<br>107-114; 116: NNS<br>107-113: NHT/RVK<br>107-109; 114-115: NHT/RVK<br>107-117: NHT/RVK	2.1E+10	87%
		IGKV3-20	30; 31; 32: RVK/DMT	56; 66**: RVK/DMT	107-113: NNS<br>107-113; 115: NNS<br>107-114; 116: NNS<br>107-113: NHT/RVK<br>107-109; 114-115: NHT/RVK<br>107-117: NHT/RVK		
		IGKV4-1	32; 33; 35: RVK/DMT	56; 66**: RVK/DMT	107-113: NNS<br>107-113; 115: NNS<br>107-114; 116: NNS<br>107-113: NHT/RVK<br>107-109; 114-115: NHT/RVK<br>107-117: NHT/RVK		
**23KN**	HV3-23-A	Naturally occurring*	Naturally occurring*	Naturally occurring*	Naturally occurring*	3.6E+09	75%

**: FR3

Name	Fixed VH	VL germlines	Position (IMGT): codon randomization CDRL1	CDRL2	CDRL3	Size	In frame sequences
**23L3**	HV3-23-A	IGLV1-40; IGLV 1-44; IGLV 1-51; IGLV 2-14; IGLV 6-57	Germ.	Germ.	109-113: NNS<br>109-114; 116: NNS<br>109-116: NNS	2.4E+09	95%
**23L13**	HV3-23-A	IGLV 1-44; IGLV 1-51; IGLV 6-57	29; 31 to 34: RVK/DMT	Germ.	109-113: NNS<br>109-114; 116: NNS<br>109-116: NNS<br>109-113: NHT/RVK<br>109-117: NHT/RVK	2.5E+09	89%
		IGLV1-40; IGLV2-14	29; 31 to 35: RVK/DMT	Germ.	109-113: NNS<br>109-114; 116: NNS<br>109-116: NNS<br>109-113: NHT/RVK<br>109-117: NHT/RVK		
**23L123**	HV3-23-A	IGLV1-40; IGLV 1-44; IGLV 1-51; IGLV 2-14; IGLV 6-57	31; 32; 33: RVK/DMT	57; 66**<br>RVK/DMT	109-113: NNS<br>109-114; 116: NNS<br>109-116: NNS<br>109-113: NHT/RVK<br>109-117: NHT/RVK	7.2E+09	85%
**23LN**	HV3-23-A	Naturally occurring*	Naturally occurring*	Naturally occurring*	Naturally occurring*	3.6E+09	95%
**S23L**	HV3-23-B	IGLV1-40; IGLV 1-44; IGLV 1-51; IGLV 2-14; IGLV 6-57	Germ.	Germ.	109-113: NNS<br>109-114; 116: NNS<br>109-116: NNS	1.9E+08	88%
**S30L**	HV3-30-B	IGLV1-40; IGLV 1-44; IGLV 1-51; IGLV 2-14; IGLV 6-57	Germ.	Germ.	109-113: NNS<br>109-114; 116: NNS<br>109-116: NNS	1.7E+08	87%
**S48L**	HV3-48-B	IGLV 1-44; IGLV 1-51; IGLV 2-14; IGLV 6-57	Germ.	Germ.	109-113: NNS<br>109-114; 116: NNS<br>109-116: NNS	2.4E+08	92%

Supplementary Table 2. Affinities of IgG isolated from fixed VH libraries. The binding kinetics were determined on an Octet RED96 or on a Biacore 2000 (*).

Target	IgG	K_D (M)	*k*a (1/Ms)	*k*d (1/s)
**hCD47**	**IgGK1**	**1.6E-10**	**7.4E+05**	**1.2E-04**
**hCD47**	**IgGK2**	**1.4E-09**	**1.5E+06**	**2.0E-03**
**hCD47**	**IgGK3**	**9.6E-10**	**1.2E+06**	**1.2E-03**
**hCD47**	**IgGK4**	**4.4E-10**	**1.1E+06**	**4.8E-04**
**hCD47**	**IgGK6**	**1.2E-09**	**7.1E+05**	**8.2E-04**
**hCD47**	**IgGK10**	**9.6E-10**	**9.1E+05**	**8.7E-04**
**hCD47**	**IgGK15**	**1.6E-06**	**5.5E+02**	**8.6E-04**
**hCD19**	**IgG1B7**	**1.1E-06**	**1.3E+03**	**1.5E-03**
**hCD19**	**IgGL7-1**	**2.6E-07**	**3.1E+03**	**8.1E-04**
**hCD19**	**IgGL7-2**	**2.3E-08**	**5.8E+03**	**1.4E-04**
**hEpCAM**	**IgGL6-2**	**4.9E-09**	**5.0E+05**	**2.4E-03**
**hEpCAM**	**IgGL6-3**	**1.8E-09**	**2.4E+05**	**4.4E-04**
**hEpCAM**	**IgGL6-4**	**2.8E-09**	**7.0E+05**	**1.9E-03**
**hEpCAM**	**IgGL6-5**	**1.7E-09**	**2.7E+05**	**4.5E-04**
**hEpCAM**	**IgGL6-6**	**3.3E-09**	**4.3E+05**	**1.4E-03**
**hMSLN**	**IgGO1**	**1.1E-08**	**3.1E+05**	**3.5E-03**
**hMSLN**	**IgGO2**	**4.6E-09**	**2.4E+05**	**1.1E-03**
**hMSLN**	**IgGO3**	**6.5E-09**	**5.4E+05**	**3.5E-03**
**hGPC-3**	**IgGP1**	**1.4E-08**	**1.0E+05**	**1.4E-03**
**hFOLR1 ***	**IgGL8-1**	**8.6E-07**	**2.2E+04**	**1.9E-02**
**hFOLR1 ***	**IgGL8-2**	**3.8E-07**	**4.7E+04**	**1.8E-02**
**hFOLR1 ***	**IgGL8-3**	**1.1E-06**	**5.1E+03**	**5.8E-03**
**hFOLR1 ***	**IgGL8-4**	**7.4E-08**	**1.9E+04**	**1.4E-03**
**hEGFR ***	**IgGN1-1**	**5.0E-09**	**2.4E+04**	**1.2E-04**
**hEGFR ***	**IgGN1-2**	**2.1E-09**	**5.3E+04**	**1.1E-04**
**hEGFR ***	**IgGN1-3**	**6.9E-09**	**1.6E+04**	**1.1E-04**
**hEGFR ***	**IgGN1-4**	**2.2E-09**	**4.1E+04**	**9.1E-05**

Supplementary Table 3. κλ-bodies generated for this study. The specificity of each bispecific arm and the identity of the corresponding IgG are indicated. The productivity observed in transient transfections of PEAK cells and the percentage of κλ-body after the IgG-CH1 Capture Select affinity chromatography step as well as the percentage of aggregates measured by SEC-HPLC in the final purified κλ-body are indicated; (nd, not determined).

κλ-body	Specificity κ arm	Specificity λ arm	IgGκ	IgGλ	Productivity (μg/mL)	% κλ-body	% aggregates
**H2-1H1-1**	IL6R	INFg	H2-1	H1-1	nd	nd	nd
**H2-1H2-2**	IL6R	IL6R	H2-1	H2-2	nd	nd	nd
**H3-1H1-1**	NusA	INFg	H3-1	H1-1	nd	nd	nd
**K1L6-1**	hCD47	hEpCam	K1	L6-1	26	24	3.5
**K2L6-1**	hCD47	hEpCam	K2	L6-1	20	22	3.4
**K3L6-1**	hCD47	hEpCam	K3	L6-1	29	13	2.4
**K4L6-1**	hCD47	hEpCam	K4	L6-1	20	21	3.4
**K5L6-1**	hCD47	hEpCam	K5	L6-1	26	17	2.3
**K6L6-1**	hCD47	hEpCam	K6	L6-1	32	24	5.3
**K7L6-1**	hCD47	hEpCam	K7	L6-1	21	17	5.5
**L6-2K8**	hEpCam	hCD47	L6-2	K8	19	nd	nd
**L6-2K9**	hEpCam	hCD47	L6-2	K9	22	29	0.9
**L6-2K10**	hEpCam	hCD47	L6-2	K10	16	nd	nd
**K1L7-1**	hCD47	hCD19	K1	L7-1	52	23	1.9
**K2L7-1**	hCD47	hCD19	K2	L7-1	47	28	1.0
**K3L7-1**	hCD47	hCD19	K3	L7-1	11	31	1.6
**K4L7-1**	hCD47	hCD19	K4	L7-1	13	47	1.2
**K5L7-1**	hCD47	hCD19	K5	L7-1	40	23	1.6
**K6L7-1**	hCD47	hCD19	K6	L7-1	51	18	2.0
**K7L7-1**	hCD47	hCD19	K7	L7-1	12	15	2.4
**L6-3L3-1**	hEpCam	hCD3	L6-3	L3-1	21	13	0.8
**L6-4L3-1**	hEpCam	hCD3	L6-4	L3-1	22	15	0.9
**L6-5L3-1**	hEpCam	hCD3	L6-5	L3-1	32	17	0.9
**L6-2L7-1**	hEpCam	hCD19	L6-2	L7-1	15	24	3.0
**K1L6-6**	hCD47	hEpCam	K1	L6-6	18	34	3.9
**K2L6-6**	hCD47	hEpCam	K2	L6-6	17	24	1.8
**K3L6-6**	hCD47	hEpCam	K3	L6-6	17	32	1.7
**K4L6-6**	hCD47	hEpCam	K4	L6-6	16	30	1.5
**K5L6-6**	hCD47	hEpCam	K5	L6-6	19	24	2.1
**K6L6-6**	hCD47	hEpCam	K6	L6-6	17	29	2.5
**K7L6-6**	hCD47	hEpCam	K7	L6-6	19	22	2.5
**K11L6-1**	hCD47	hEpCam	K11	L6-1	23	14	4.7
**K12L6-1**	hCD47	hEpCam	K12	L6-1	18	23	4.0
**K13L6-1**	hCD47	hEpCam	K13	L6-1	14	23	2.3
**K14L6-1**	hCD47	hEpCam	K14	L6-1	34	10	10.6
**K15L6-1**	hCD47	hEpCam	K15	L6-1	16	25	2.4
**K11L7-1**	hCD47	hCD19	K11	L7-1	16	11	3.3

**K12L7-1**	hCD47	hCD19	K12	L7-1	16	20	2.2
**K13L7-1**	hCD47	hCD19	K13	L7-1	16	37	2.0
**K14L7-1**	hCD47	hCD19	K14	L7-1	13	9	4.1
**K15L7-1**	hCD47	hCD19	K15	L7-1	17	34	1.6
**K11L7-2**	hCD47	hCD19	K11	L7-2	13	13	3.8
**K12L7-2**	hCD47	hCD19	K12	L7-2	18	16	3.0
**K2L7-2**	hCD47	hCD19	K2	L7-2	20	23	1.5
**K3L7-2**	hCD47	hCD19	K3	L7-2	19	47	1.6
**K13L7-2**	hCD47	hCD19	K13	L7-2	18	35	12
**K15L7-2**	hCD47	hCD19	K15	L7-2	18	33	1.7
**K14L7-2**	hCD47	hCD19	K14	L7-2	18	6	7.0
**K16L7-2**	hCD47	hCD19	K16	L7-2	8	51	1.7
**K16L6-1**	hCD47	hEpCam	K16	L6-1	16	17	2.5
**L6-2L7-2**	hEpCam	hCD19	L6-2	L7-2	29	21	2.7

Supplementary Table 4. Receptor density on Raji vesus DS-1 cell lines.

	Number of receptor/cell	
	**CD47**	**CD19**
**Raji**	67’195	72’190
**DS-1**	136’000	Not expressed

Supplementary Table 5. Screening strategy for the generation of recombinant CHO cell lines transfected with expression vectors for κλ-body generation (κλ triple gene cassette) or monospecific IgG controls (κκ or λλ double gene cassettes).

Step	H2-1H1-1			H2-1H2-2 (κλ only)
	κκ	κλ	λλ	
Number of 96-well transfection plates	10	10	10	10
Number of colonies identified in 96-well plates	106	111	32	82
Number of cell lines progressed into 24-well plates	20	111	20	82
Number of cell lines progressed into productivity assessment in batch shake flask cultures	10	25	9	10

Supplementary Table 6. Pharmacokinetics of monospecific κκ and λλ and bispecific κλ-bodies after i.v. administration in mice

Data are from 3 mice per time point, 5 mg/kg i.v. injection

	hIgG1κ	hIgG1λ	H2-1H1-1	K15L7-2
CL (mL/kg/day)	3.06	3.79	4.44	6.14
$T_{1/2}$ terminal (day)	19.7	20.1	17.8	16.8
